# Development and validation of a machine learning-based predictive model for carotid plaque in type 2 diabetes

**DOI:** 10.3389/fcvm.2026.1801899

**Published:** 2026-06-12

**Authors:** Yuwei Xing, Lili Zhang, Qianqian Zhao

**Affiliations:** Department of Endocrinology, The Second Hospital of Shijiazhuang, Shijiazhuang, China

**Keywords:** carotid plaque, machine learning, primary care, SHAP, type 2 diabetes mellitus

## Abstract

**Introduction:**

Carotid plaque is a critical risk factor for cardiovascular disease and reflects the extent of the atherosclerotic burden. Compared with non-diabetic individuals, patients with type 2 diabetes mellitus (T2DM) have an elevated likelihood of developing carotid plaques. Consequently, building predictive models tailored to this high-risk population holds significant clinical value for early prevention and management of cardiovascular events.

**Methods:**

A total of 2,288 patients were included in this study, 1,716 (75.0%) of whom had plaques detected using ultrasound. Baseline data, including demographic characteristics, medical history, and laboratory indicators, were collected, and seven machine-learning algorithms were applied to establish the prediction model. Feature importance was quantified and presented using Shapley Additive Explanations (SHAP). The performance of the model was evaluated using indicators such as area under the curve (AUC-ROC), sensitivity, and specificity.

**Results:**

The study showed that The logistic regression model performed the best in terms of discrimination, calibration, and clinical utility. Through SHAP interpretability analysis, key risk factors such as age, body mass index, glycated hemoglobin, history of hypertension, monocyte count, neutrophil percentage, red blood cell count, sex, estimated glomerular filtration rate, and statin use were identified. This study demonstrated that an effective risk-prediction model can be established using conventional clinical variables and machine learning.

**Conclusions:**

The developed predictive model can help primary care providers detect patients at heightened risk of carotid atherosclerotic plaques, enabling the delivery of targeted preventive strategies and ultimately improving clinical outcomes.

## Graphical Abstract

**Figure:**
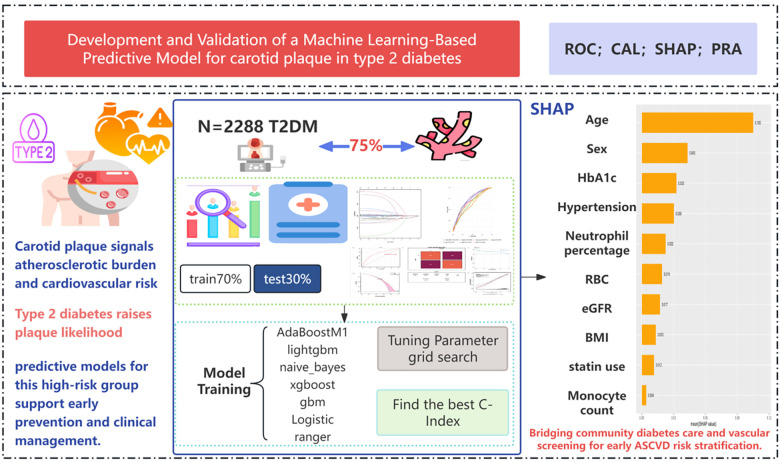

## 1 Introduction

Carotid artery plaques are a significant manifestation of atherosclerosis and one of the main risk factors for stroke and cardiovascular events (1). Patients with type 2 diabetes mellitus (T2DM) have an accelerated process of atherosclerosis due to long-term hyperglycemia, dyslipidemia, inflammatory responses, and other factors, and the incidence of carotid artery plaques is significantly higher than that in non-diabetic populations (2). Studies have shown that T2DM patients not only have a higher incidence of carotid artery plaques (3) but also that the nature of the plaques is often more unstable and prone to rupture and thrombosis, leading to serious clinical events. Therefore, early identification and assessment of carotid artery plaque risk in T2DM patients are crucial for preventing cardiovascular and cerebrovascular complications.

Traditional risk assessment methods (such as the Framingham score) and single biomarkers have limitations in predicting carotid plaques in patients with T2DM because they cannot capture the complexity of the disease (4). Moreover, within China's primary healthcare facilities, the inadequate availability of carotid ultrasound resources and specialized personnel pose significant barriers to the early detection and risk stratification of carotid plaque formation. Addressing this limitation necessitates the development of risk stratification tools.

Recent machine learning (ML) advances have improved clinical prediction, but their “black-box” opacity limits interpretability and clinical actionability (5). Shapley Additive exPlanations (SHAP), a model-agnostic technique grounded in Shapley values, quantifies each feature's contribution to individual predictions, decomposing outputs into additive attributions and thereby providing both local and global explanations to enhance transparency (6, 7).

Few studies have specifically developed and externally validated machine-learning models to predict carotid plaque in type 2 diabetes. Although SHAP has improved transparency in other clinical areas, its use for diabetes-related cardiovascular risk prediction is limited. We developed and internally validated a machine-learning model using routinely available clinical variables to identify the presence of carotid plaque (prevalent plaque) among patients with type 2 diabetes, then applied SHAP to quantify each feature's marginal contribution and interaction effects on predicted plaque risk.

## 2 Methods

### 2.1 Study participants

This study was a cross-sectional analysis conducted on patients with type 2 diabetes who visited a single tertiary medical center.The study population consisted of diabetic patients who visited the Second Hospital of Shijiazhuang City between January 2024 and December 2025. The Second Hospital of Shijiazhuang is a comprehensive tertiary hospital and the largest diabetes-specialized medical center in North China, located in Shijiazhuang of Hebei province, which serves as a regional referral hub for endocrine disorders. Eligibility required participants to be ≥18 years old and to meet ADA criteria for type 2 diabetes (8), demonstrated by one or more of the following: fasting plasma glucose ≥7.0 mmol/L; HbA1c ≥6.5%; or a documented T2DM diagnosis by an endocrinologist, with the individual currently on lifestyle modification and/or glucose-lowering therapy. Subjects were excluded if they had (1) undergone prior carotid intervention or had major neck anatomical alterations that would compromise ultrasound evaluation; (2) undergone neck radiotherapy in the last 3 months; (3) experienced an acute carotid-related cerebrovascular event within 6 months; (4) diagnosis of diabetes other than T2DM (including type 1, gestational, monogenic, post-surgical, or endocrine secondary diabetes); (5) recent treatment with potentially confounding medications, such as systemic glucocorticoids at prednisone-equivalent doses ≥20 mg/day for ≥2 weeks or any immunosuppressive/immunomodulatory agents within 3 months; (6) participated in interventional clinical trials involving agents expected to alter lipid metabolism, systemic inflammation, or vascular function; (7) severe comorbidities (e.g., terminal malignancy with life expectancy <6 months or other end-stage diseases likely to interfere with study procedures); and (8) inability to obtain or complete standardized carotid ultrasound imaging of sufficient quality for Intima-Media Thickness (IMT) or plaque analysis.

The study protocol was approved by the hospital's Ethics Committee (approval no. SEY202011117). Because this was a retrospective analysis, the committee waived the requirement for written informed consent. All personal identifiers were removed and the data were anonymized in accordance with the ethical principles of the Declaration of Helsinki.

### 2.2 Potential risk features and outcomes

We performed a systematic review of the current literature to compile candidate risk factors for carotid artery plaque and then narrowed the list to 32 variables by including only factors with corresponding analyzable data available within our study (Supplementary Table S1).

The candidate predictor variables included in this study were grouped into the following categories: Demographics (Sex, Age); Lifestyle Habits (Smoking History, Drinking History); Clinical Assessments and Vital Signs (SBP, Systolic Blood Pressure; DBP, Diastolic Blood Pressure; BMI, Body Mass Index); Medical History (Hypertension; DM Duration, Diabetes Mellitus Duration; Family History of Diabetes; Family History of Hypertension; Statin Use); and Laboratory Examinations (HbA1c, Glycated Hemoglobin; WBC, White Blood Cell Count; Lymphocyte Count; Neutrophil Count; Neutrophil Percentage; Monocyte Count; RBC, Red Blood Cell Count; HGB, Hemoglobin; RDW-SD, Red Cell Distribution Width-Standard Deviation; PLT, Platelet Count; PDW, Platelet Distribution Width; BUN, Blood Urea Nitrogen; CREA, Serum Creatinine; UA, Uric Acid; eGFR, Estimated Glomerular Filtration Rate; TG, Triglycerides; CHOL, Total Cholesterol; HDL, High-Density Lipoprotein Cholesterol; LDL, Low-Density Lipoprotein Cholesterol; hs-CRP, High-sensitivity C-Reactive Protein).

The outcome measure was the presence or absence of carotid artery plaque at baseline visits. Ultrasound scanning protocol.

A high-definition color Doppler ultrasound system (EPIQ Elite) was used to thoroughly assess both the carotid arteries. The scan covered the common carotid arteries on each side (CCA, approximately 1–2 cm before bifurcation), carotid bifurcation and bulb, internal carotid artery (ICA, approximately 2 cm beyond the bulb), and the external carotid artery (ECA) when clinically required. The examinations were conducted by vascular ultrasound physicians with appropriate professional certifications and substantial experience. Before starting the study, a standardized scanning and data collection protocol was adopted, and a detailed measurement and operation manual was prepared to ensure uniformity in technique and interpretation.

#### 2.2.1 Definition and evaluation criteria of plaques

Carotid atherosclerotic plaque definitions follow the Mannheim IMT and plaque consensus (9). A plaque is present if any segment shows a focal structure protruding into the lumen, focal IMT ≥50% thicker than adjacent normal wall, or focal IMT >1.5 mm. IMT was measured on longitudinal images, taking the maximal thickness from intima–lumen to media–adventitia according to this center's protocol. Imaging required completion of bilateral carotid color Doppler; two independent sonographers judged adequacy, and only images accepted by both were used. Disagreements were resolved by a senior third sonographer.

### 2.3 Statistical analysis

The dataset was randomly divided into a development/validation set (70%) and an internal hold-out test set (30%) using stratified sampling by outcome. Temporal validation was performed using temporally distinct data from the same institution (January–February 2026, *n* = 135) without model recalibration. To address the inherent trade-off between sensitivity and specificity in screening contexts, we performed a systematic threshold optimization analysis beyond the conventional default of 0.5. We evaluated multiple candidate cutoffs (0.5 and 0.7) using the temporal validation data, with selection guided by the Youden index (*J* = sensitivity + specificity−1), clinical relevance, and the prioritization of minimizing missed diagnoses in a screening setting. Performance metrics, including sensitivity, specificity, positive predictive value (PPV), negative predictive value (NPV), and the Matthews correlation coefficient (MCC), were computed with 95% confidence intervals (CIs). The decision curve analysis (DCA) was additionally used to assess net benefit across threshold probabilities, accounting for the relative clinical harm of false-negative vs. false-positive results. For continuous variables, normality was assessed using appropriate tests. Normally distributed variables were presented as mean ± standard deviation and were compared using an independent sample *t*-test. Non-normally distributed variables are shown as medians (interquartile ranges) and were compared using the Mann–Whitney *U*-test. Categorical variables were reported as frequencies (percentages) and compared using either the chi-squared test or Fisher's exact test. All statistical tests were two-tailed, and a *p*-value < 0.05 was considered statistically significant.

### 2.4 Feature preprocessing

Prior to modeling, the candidate features were preprocessed to enhance the performance and stability of the model. Missing data: Missing values were imputed using the random-forest algorithm. Categorical Features: For features with more than two categories, one-hot encoding was applied to convert them into numerical format. Continuous Features with Outliers: Binning was performed on features with significant outliers to reduce their influence on the model. Feature preprocessing was integrated into the *pipeline* functions in conjunction with the algorithms to ensure that the validation set data underwent the same processing.

### 2.5 Feature selection

First, we excluded the variables with zero variance and excessive collinearity. The Spearman's rank correlation test was used to detect collinearity. The feature with the strongest association with the outcome was considered to be when the correlation coefficient exceeded 0.8.

Following the initial filtering, 32 variables were subjected to the Least Absolute Shrinkage and Selection Operator (Lasso) regression analysis. This method applies L1 regularization penalties to the regression coefficients, promoting sparsity by shrinking the less important coefficients to zero, thereby achieving simultaneous variable selection and model simplification. The LASSO tuning parameter (*λ*) was selected by ten-fold cross-validation (cv.glmnet), examining both *λ*_min (value that minimizes the mean cross-validated error) and *λ*_1se (largest *λ* within one standard error for a more parsimonious model). We selected *λ*_min to maximize predictive accuracy, which produced 10 non-zero coefficients; these ten variables were retained as candidate predictors for subsequent modelling. Predictors were standardized and LASSO was implemented with *α* = 1 (glmnet default).

To prevent data leakage, all preprocessing and feature selection were confined to the training set within the cross-validation loop and then applied to the internal test set, ensuring unbiased performance evaluation.

### 2.6 Model construction and validation

#### 2.6.1 Algorithm selection

Seven diverse machine learning algorithms were implemented to provide a comprehensive comparative analysis: AdaBoostM1(Adaptive Boosting M1), Gradient Boosting Machine (gbm), Light Gradient Boosting Machine (lightgbm), Logistic Regression (logistic), Naïve Bayes(naive_ Bayes), Random Forest (ranger), and extreme Gradient Boosting (XGBoost). These methods comprise ensemble/tree-based algorithms (AdaBoostM1, gbm, LightGBM, ranger, and XGBoost), a linear model (Logistic Regression), and a probabilistic classifier (Naïve Bayes), thereby enabling multidimensional data analysis and robust performance evaluation.

#### 2.6.2 Handling of class imbalance

To address the issue of class imbalance, in the model training process, the samples are weighted according to the inverse of the class frequency (class balance), so that the contribution of each class to the loss function is inversely proportional to the number of samples. The weighting is only applied to the training folds.

#### 2.6.3 Performance evaluation

Model performance was assessed using a multi-metric framework, including Classification Metrics: Sensitivity, specificity, F1 score (harmonic mean of precision and recall), and overall accuracy, which were calculated to assess the classification performance across different aspects.

Discriminative Capability: Area under the receiver operating characteristic curve (AUROC) evaluated the model's ability to distinguish between positive and negative cases across various decision thresholds. The precision-recall curve (AUPRC) specifically focuses on positive case identification, which is particularly important for imbalanced datasets.

Model Calibration: Calibration curves were used to assess the concordance between predicted probabilities and observed outcomes, ensuring reliable probability estimates for clinical decision making.

To make a fair comparison between the algorithms, the Logistic Regression model was selected as the optimal model for the subsequent optimization and implementation.

#### 2.6.4 Overfitting control

To mitigate overfitting, a dual validation strategy was employed: stratified five-fold cross-validation, in which the data were divided into five subsets, where one subset served as the test set, and the remaining four were used for training. The performance metrics were averaged over five folds to evaluate model stability and predictive performance. Independent Test Set Validation: The optimized model was applied to an independent test set to assess its generalizability further.

#### 2.6.5 Model interpretation

To investigate the contribution of each individual feature to the prediction of carotid plaque risk, we employed Kerne SHAP to compute SHAP values from the **Logistic Regression** model (10, 11). The SHAP values, representing the logarithmic probability of individual contributions, were visualized using the R package, shapviz (v.0.9.6). SHAP facilitates both local and global interpretability. To investigate the contribution of individual features to the model's predictions of prevalent carotid plaque, we applied Kernel SHAP to compute SHAP values from the logistic regression model. SHAP values quantify each feature's contribution to the model-predicted probability in this dataset and should be interpreted as model-based associations rather than causal effects.To interpret the feature importance in the optimal model, we calculated the importance of the feature rankings to quantify the contribution of each feature to the predictions of prevalent carotid plaque. The model performance and interpretation were evaluated using the DALEX package (v2.4.3).

#### 2.6.6 Web application

An interactive web application was developed using R shiny (https://www.shinyapps.io/) to enable real-time prediction of carotid plaque risk by anesthesiologists.

**Figure 1:**
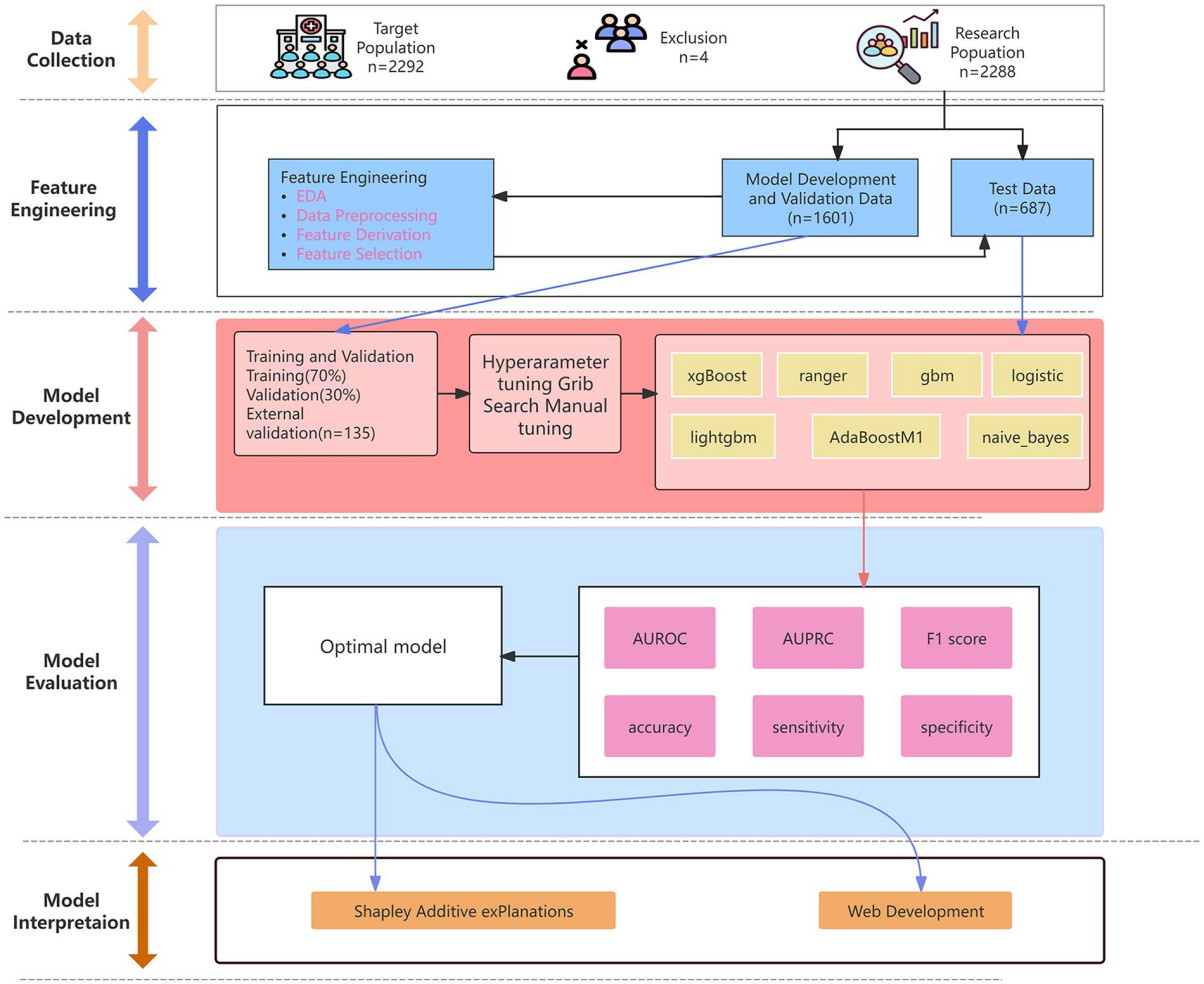
Flowchart of machine learning model development for predicting carotid plaque in patients with type 2 diabetes. The schematic outlines the key stages from data collection to model deployment. **Data Collection and Feature Engineering:** Initial cohort included 2,292 patients. After excluding four patients, 2,288 were eligible. Exploratory data analysis (EDA), data preprocessing, feature derivation, and feature selection were performed to prepare the feature set. **Data Splitting:** The research population was divided into Model Development and Validation Data (*n* = 1,601), and independent Test Data (*n* = 687). **Model Development:** The development data were further split into training (70%) and validation (30%) sets. Hyperparameter tuning was conducted via a grid search and manual adjustment. **Model Evaluation:** The optimal model was evaluated using a test cohort. Performance metrics included the area under the receiver operating characteristic curve (AUROC), area under the precision-recall curve (AUPRC), F1 score, accuracy, sensitivity, and specificity. **Model Interpretation and Deployment:** Model predictions were interpreted using the (SHAP). A web-based tool has been developed for clinical use.

All data processing, model development, and validation procedures were performed using R, version 4.2.2. The analytical pipeline utilizes multiple specialized libraries, including mlr3 (machine learning framework), mlr3learners (algorithm implementations), mlr3extralearners (additional algorithms), mlr3tuning (hyperparameter optimization), mlr3pipelines (preprocessing workflows), mlr3filters, and mlr3fselect (feature selection) (Figure 1). This comprehensive toolkit ensured robust and reproducible model development following best practices in the machine learning methodology. The deployed prediction tool is designated as Version 1.0, with future model updates planned to be implemented under the same uniform resource locator (URL) to ensure seamless accessibility.

## 3 Results

### 3.1 Patient characteristics

A total of 2,288 participants were included (mean age 63.0 ± 11.6 years; range 16–91), with females accounting for 46.3% of the sample. The dataset was randomly partitioned into a development/validation set (*n* = 1,601) for model building and internal validation and an independent test set (*n* = 687) for external evaluation. The baseline characteristics are provided in Supplementary Table S2, except for the neutrophil percentage. No statistically significant differences were observed between the two subsets, supporting the comparability of the groups in the subsequent predictive analyses.

### 3.2 Feature selection

**Figure 2:**
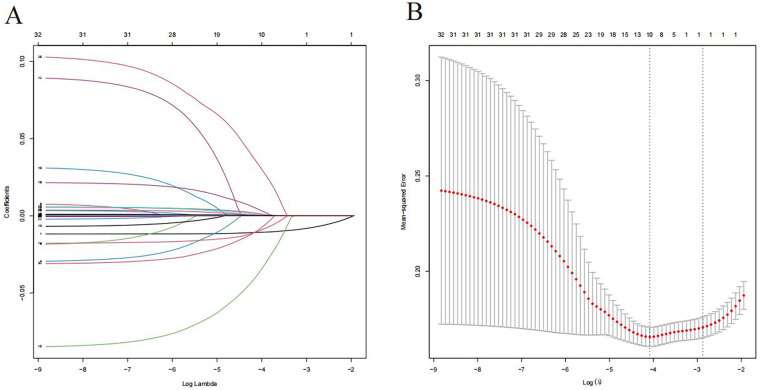
Lasso regression-based feature selection. **(A)** Variation characteristics of variable coefficients; **(B)** The process of selecting the optimal value of the parameter *λ* in the lasso regression model is carried out by the cross-validation method.

Prior to the model development, we implemented a structured feature selection pipeline to improve model applicability, reduce redundancy, and enhance interpretability. None of the predictors exhibited a zero variance. Multicollinearity was evaluated using Pearson correlation coefficients and variance inflation factors with pre-specified thresholds (|r| > 0.8 or VIF >10); no variables met the exclusion criteria, suggesting minimal linear redundancy among the candidates. LASSO (least absolute shrinkage and selection operator) regression—chosen for its simultaneous selection and regularization properties—identified ten predictors significantly associated with carotid plaque: age, body mass index (BMI), HbA1c, hypertension, monocyte count, neutrophil percentage, red blood cell count (RBC), sex, estimated glomerular filtration rate (eGFR), and statin use (Figures 2A,B). The correlation matrices prior to and following the feature selection are shown in Supplementary Figures S1, S2.

### 3.3 Model evaluation and benchmarking

**Figure 3:**
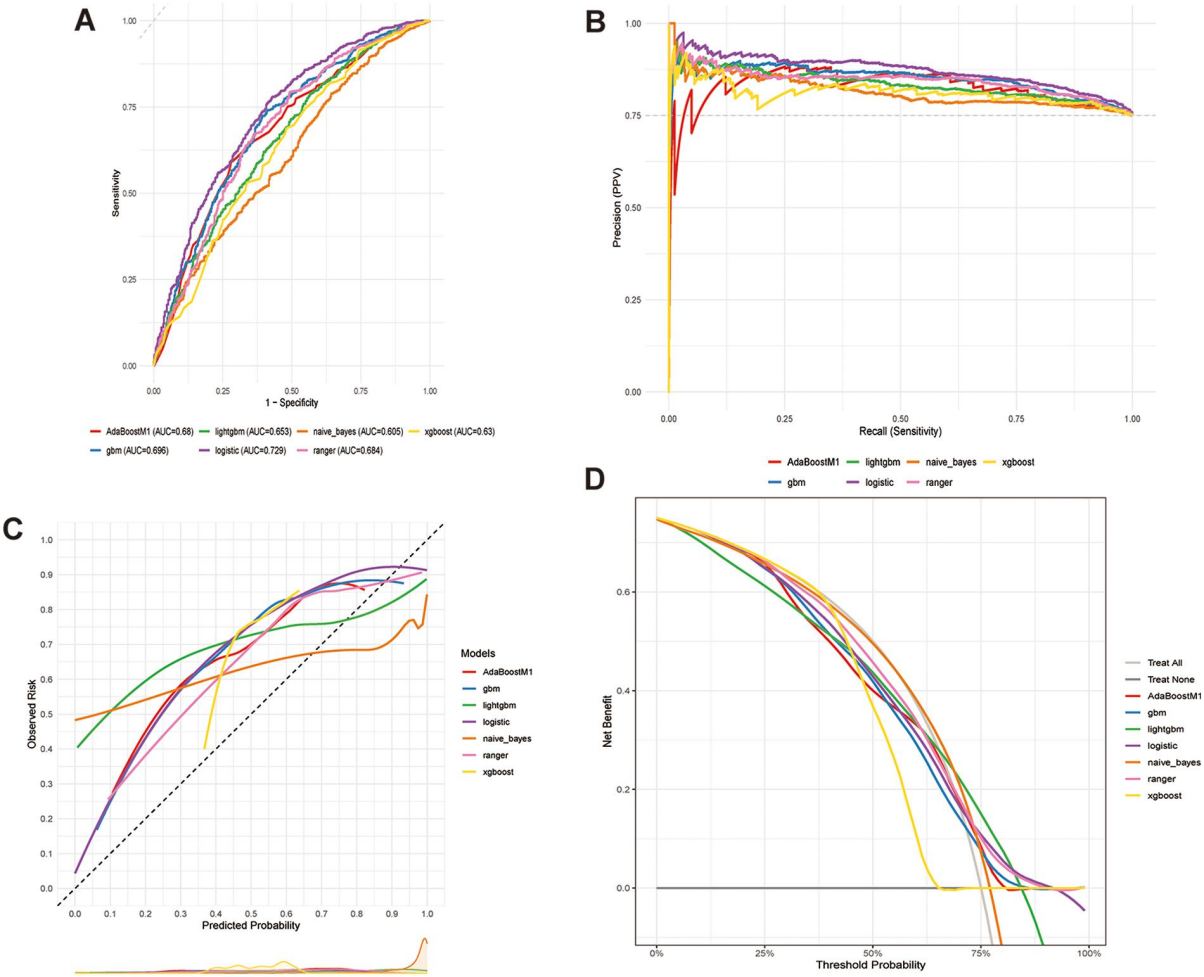
Performance comparison of the ML models of benchmarking on the development/validation sets. **(A)** Receiver operating characteristic curve (ROC). **(B)** Precision-recall curve (PRC). **(C)** Calibration curve (CAL). **(D)** Decision curve analysis (DCA). AdaBoostM1(Adaptive Boosting M1), Gradient Boosting Machine (gbm), light-gradient boosting machine (lightgbm), Logistic Regression (logistic), Naïve Bayes(naive_ Bayes), Random Forest (ranger), and extreme Gradient Boosting (xgboost).

**Figure 4:**
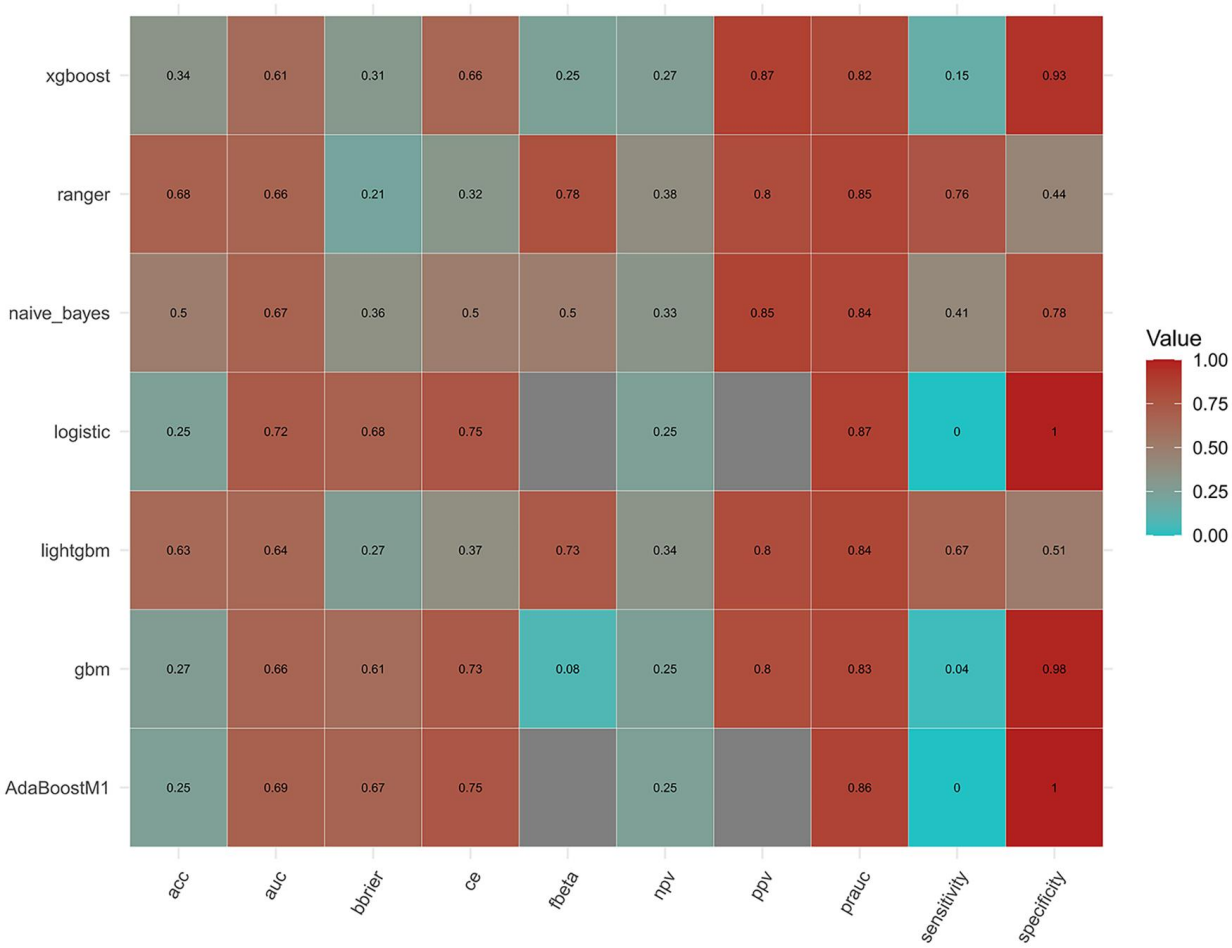
Performance heatmap of machine learning model benchmarking on development/validation sets. The models evaluated include AdaBoostM1(Adaptive Boosting M1), Gradient Boosting Machine (gbm), Light Gradient Boosting Machine (lightgbm), Logistic Regression (logistic), Naïve Bayes(naive_Bayes), Random Forest (ranger), and extreme Gradient Boosting (XGBoost). Performance metrics: Area Under the Curve (AUC), accuracy (ACC), Brier Score (Brier score), F-Beta (F-Beta Score), Precision-Recall Area Under the Curve (PRAUC), sensitivity, specificity, Negative Predictive Value (NPV), and Positive Predictive Value (PPV).

Ten variables were retained after selection. In a benchmark of seven algorithms, logistic regression attained the highest Receiver Operating Characteristic (ROC) AUC (0.729) and, by five-fold cross-validation, showed an accuracy of 0.68, a sensitivity of 0.69, a specificity of 0.65, and an F1 of 0.766 (Figures 3A, 4; Supplementary Table S3). However, precision-recall, calibration, and decision-curve analyses (Figures 3B–D) favored the Logistic Regression model for clinical utility; therefore, Logistic Regression was selected for further optimization and implementation.

### 3.4 Hyperparameter optimization and performance in development/validation sets

**Figure 5:**
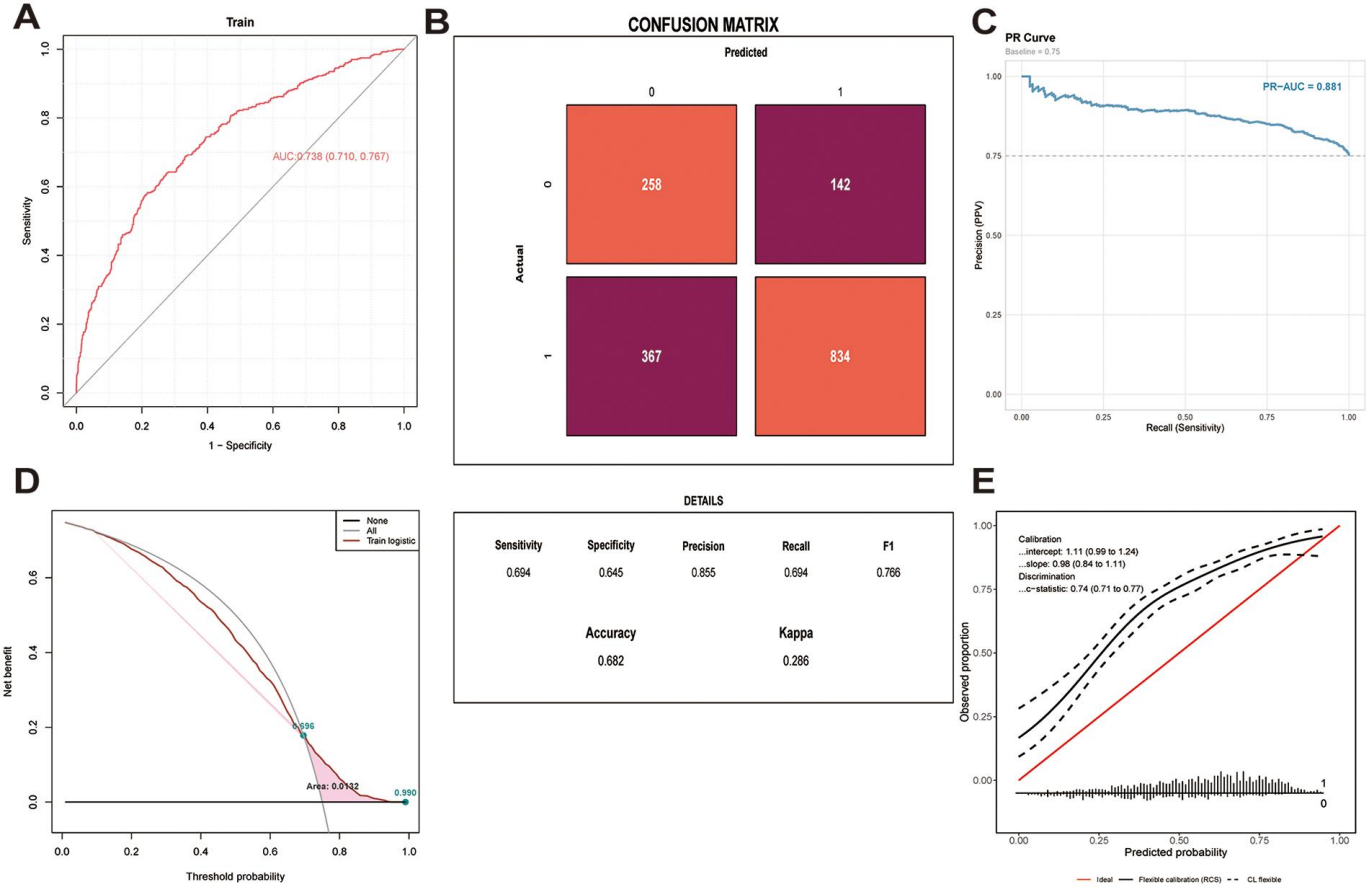
Performance of the logistic regression in the development/validation sets. **(A)** Receiver operating characteristic curve (ROC). **(B)** Confusion matrix. **(C)** Precision-recall curve (PRC). **(D)** Calibration curve (CAL). **(E)** Decision curve analysis (DCA). **(F)** Metric radar plot.

We assessed several candidate algorithms, and ultimately proceeded with a logistic regression classifier for validation. Using five-fold cross-validation of the development/validation cohort, the ROC AUC of the model increased to 0.738 (Figure 5A). The performance metrics were accuracy = 0.682, precision = 0.855, sensitivity (recall) = 0.694; specificity, 0.645; and F1 score, 0.766 (Figure 5B; Supplementary Table S5). The relatively high precision (0.855) is notable because it helps limit false-positive diagnoses. The precision–recall curve, calibration plot, and decision curve analysis(DCA) from the five-fold CV on the development/validation set are shown in Figures 5C–E. Logistic regression also showed good calibration (Brier score = 0.205), a PR AUC of 0.881, and a net benefit in DCA for threshold probabilities between 69.6% and 99.0% (DCA AUC = 0.0132), outperforming the two extreme strategies of “no treatment” and “treat all.”.

### 3.5 Test set performance

**Figure 6:**
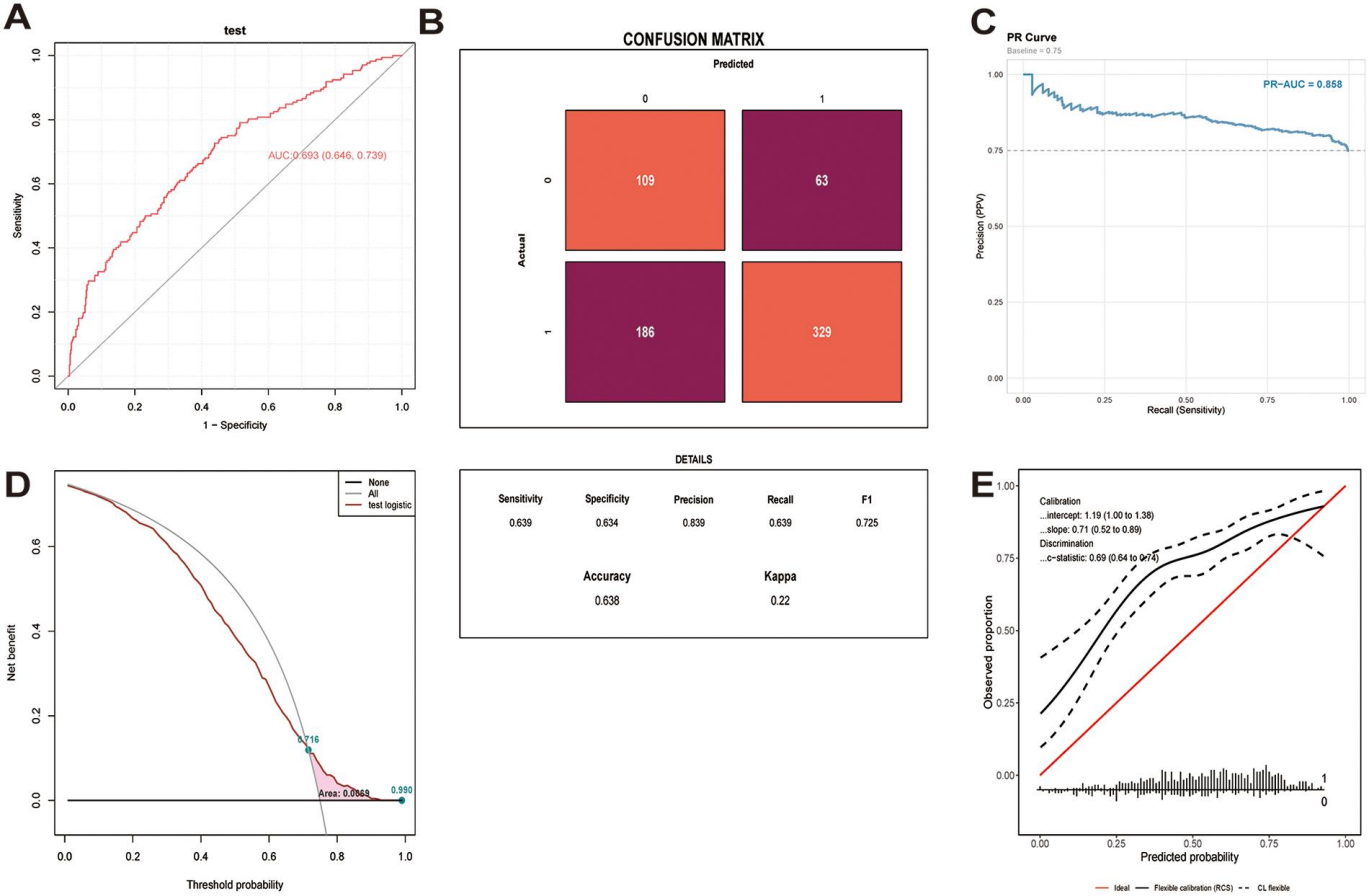
Performance of the logistic regression in the test set. **(A)** Receiver operating characteristic curve (ROC). **(B)** Confusion matrix. **(C)** Precision-recall curve (PRC). **(D)** Calibration curve (CAL). **(E)** Decision curve analysis (DCA).

For independent test data, the logistic regression classifier reproduced the performance observed during development and validation, yielding an area under the ROC curve (AUC) of 0.693 (Figure 6A). Its overall accuracy was 0.638, precision 0.839, sensitivity (recall) 0.639, specificity 0.634, and F1 score 0.725 (Figure 6B, Supplementary Table S5). Calibration curves, precision–recall plots, DCA, and radar charts for the 5-fold cross-validated test set are presented in Figures 6C–E. The summary metrics included a brier score of 0.2244, PR-AUC of 0.858, and DCA-AUC of 0.0069, with the model providing a greater net clinical benefit than the alternative strategies for threshold probabilities between 71.6% and 99%.

**Figure 7:**
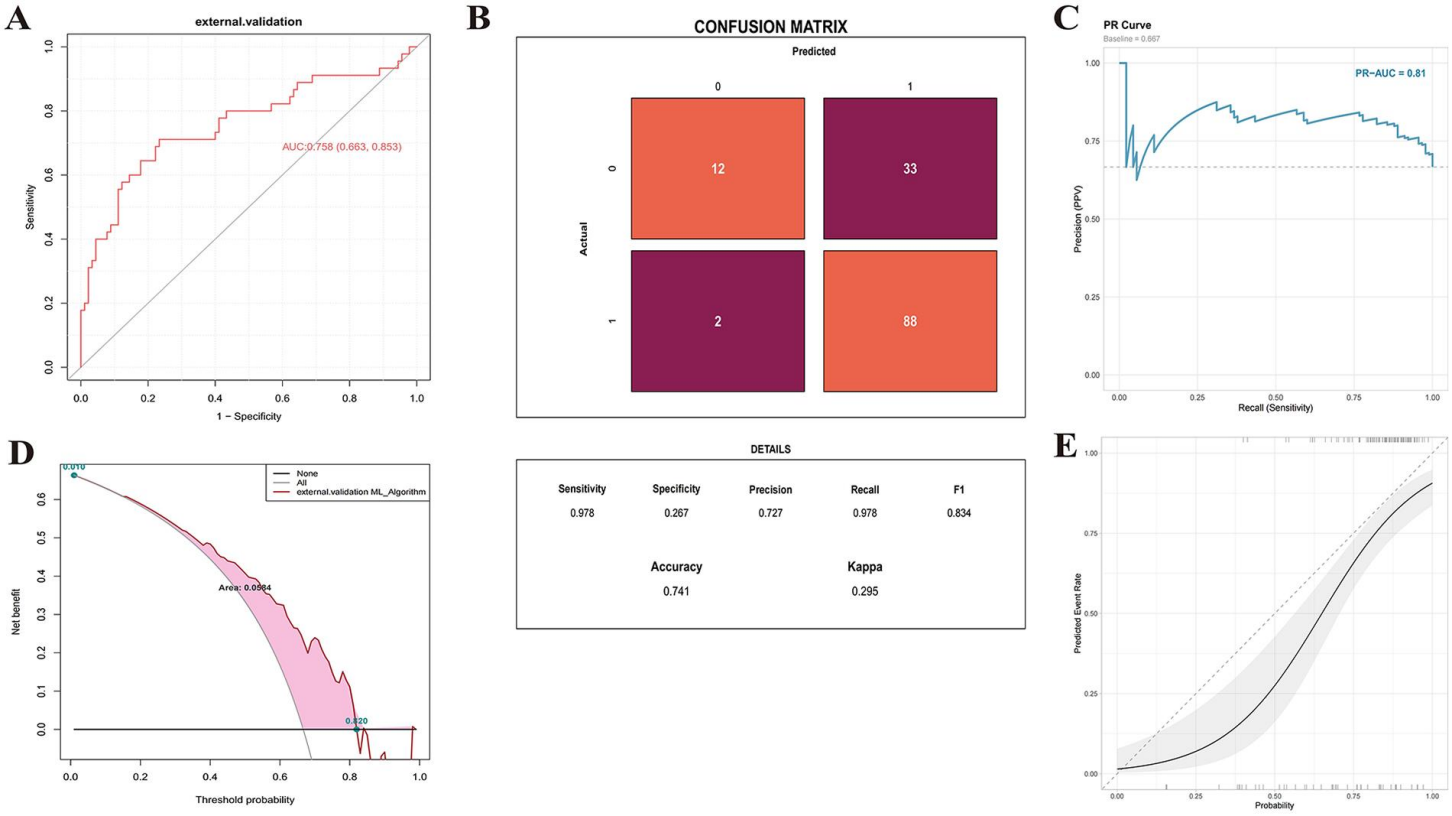
Performance of the logistic regression in the temporal validation set. **(A)** Receiver operating characteristic curve (ROC). **(B)** Confusion matrix. **(C)** Precision-recall curve (PRC). **(D)** Calibration curve (CAL). **(E)** Decision curve analysis (DCA).

Temporal validation showed that the area under the ROC curve (AUC) was 0.758 (Figure 7A). The overall accuracy was 0.741, the precision was 0.727, the sensitivity (recall rate) was 0.978, the specificity was 0.268, and the F1 score was 0.834 **(**Figure 7B). The calibration curve, precision-recall plot, and DCA of the 5-fold cross-validation test set are shown in Figures 7C–E. Summary metrics include a brilliance score of 0.183, a PR-AUC of 0.81, and a DCA-AUC of 0.0584. This model provided greater net clinical benefits than other strategies within the threshold probability range of 1% to 82%.

**Figure 8:**
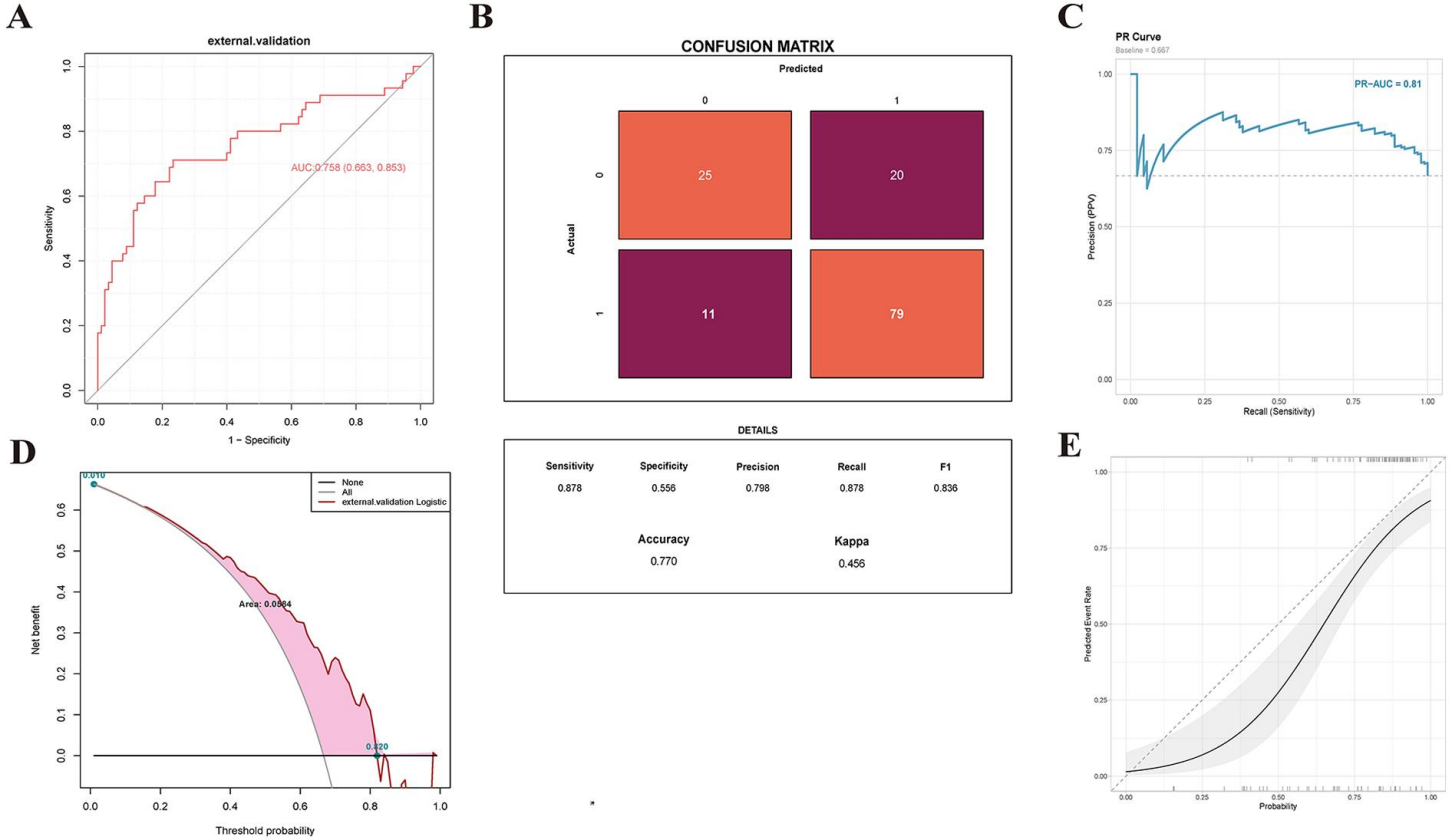
Model performance at the optimized threshold of 0.7 in temporal validation data. **(A)** Receiver operating characteristic (ROC) curve with area under the curve (AUC) of 0.758 (95% CI: 0.670–0.843). **(B)** Confusion matrix at threshold 0.7, showing 20 true negatives, 25 false positives, 11 false negatives, and 79 true positives. **(C)** Precision-recall (PR) curve with PR-AUC of 0.81 and baseline of 0.667. **(D)** Decision curve analysis demonstrating net benefit across threshold probabilities; the model (red curve) shows positive net benefit over treat-all (grey) and treat-none (black) strategies between threshold probabilities of approximately 0.10 and 0.70. **(E)** Calibration plot comparing predicted vs. observed event rates with 95% confidence bands (grey shading).

Further optimization at a threshold of 0.7 achieved a more favorable balance: sensitivity remained acceptable at 0.878 (95% CI: 0.809–0.943), while specificity improved substantially to 0.556 (95% CI: 0.416–0.690) (Supplementary Table S4, Figure 8). The PPV increased to 0.798 (95% CI: 0.719–0.872), indicating that approximately four in five flagged cases were true positives, and the NPV was 0.694 (95% CI: 0.560–0.842). The Youden index reached 0.433, and the MCC was 0.462 (95% CI: 0.316–0.605), reflecting moderate overall agreement. Balanced accuracy was 0.717 (95% CI: 0.643–0.789), and the F1-score was 0.836 (95% CI: 0.777–0.887). Notably, the ROC-AUC remained stable at 0.758 across thresholds, indicating that the model's discriminative capacity was unchanged and that threshold adjustment represents a calibration rather than a discrimination improvement.

A high PR-AUC indicated a favorable balance between precision and sensitivity for identifying the presence of carotid plaque on contemporaneous imaging, addressing class imbalance in the cross-sectional dataset.

### 3.6 Model interpretability: SHAP analysis

**Figure 9:**
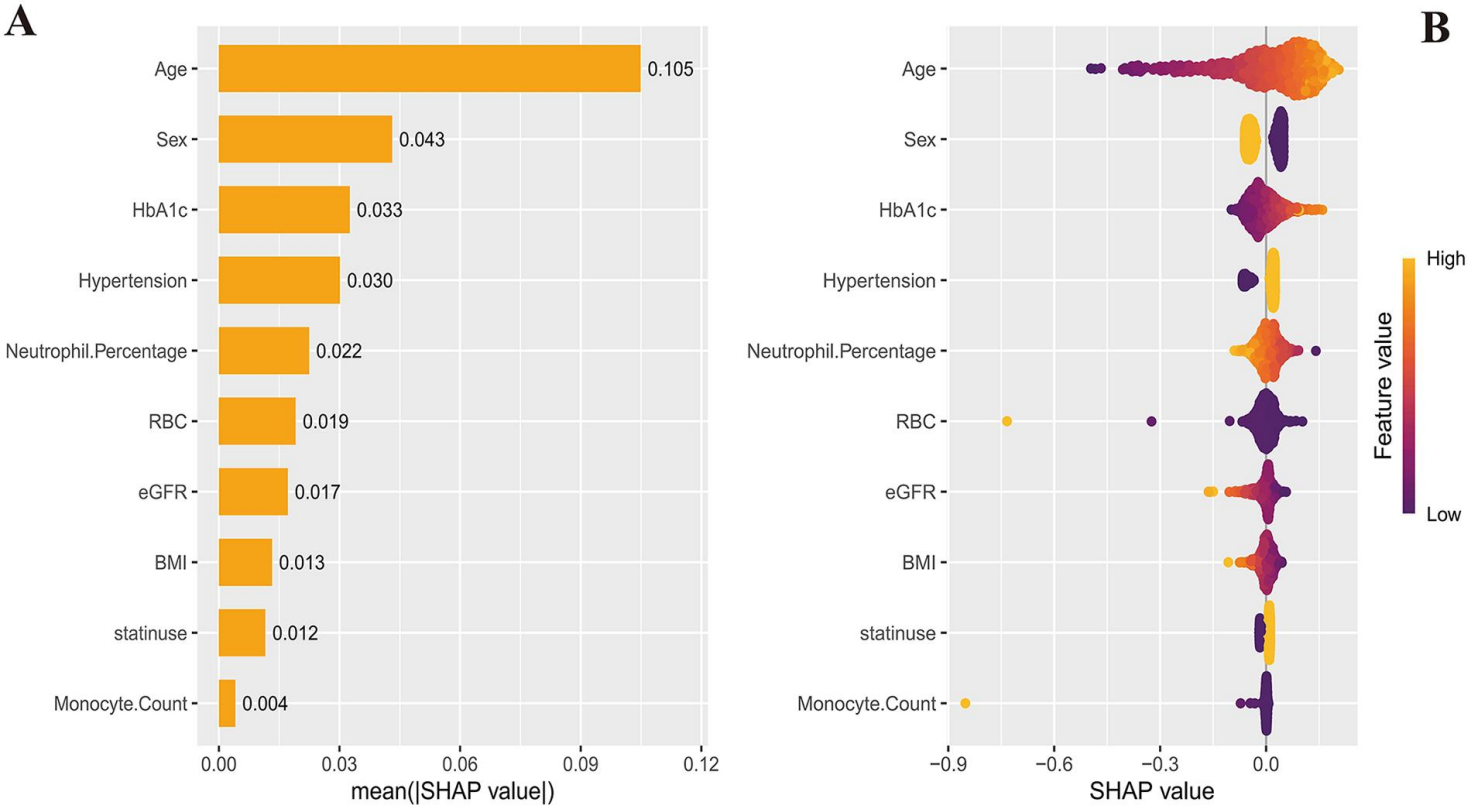
Global model explanation by the SHAP method. **(A)** SHAP summary dot plot of the Logistic Regression model. Each line represents a feature, and the *x*-axis represents SHAP values, indicating the impact of the feature on the outcome. Each dot represents an individual patient. The yellower the color, the larger the feature value; the bluer the color, the smaller the feature value. **(B)** SHAP feature importance plot of the Logistic Regression model.

Given that clinicians generally do not adopt predictive tools lacking interpretability, we employed the SHAP (Shapley Additive Explanations) method to provide global and local explanations for the final model. The global analysis revealed the overall behavior of the model, and its SHAP values are shown in Figure 9A, whereas the ranking of the feature importance is presented in Figure 9B. In the Logistic regression model, the top ten influential features were age, sex, glycated hemoglobin, presence of hypertension, neutrophil ratio, RBC count, eGFR, BMI, statin use, and monocyte count. Supplementary Figure S3 shows the relationship between the actual values of these ten variables and their corresponding SHAP values, a positive SHAP value indicates that the feature increases the presence of carotid plaque predicted probability, whereas a negative SHAP value decreases it. SHAP values indicate associations learned by the predictive model and should not be interpreted as evidence of causal effects. For instance, individuals who were older or had a history of hypertension were more likely to have positive SHAP values, thereby biasing the model's ability to predict “the presence of carotid plaques.”.

**Figure 10:**
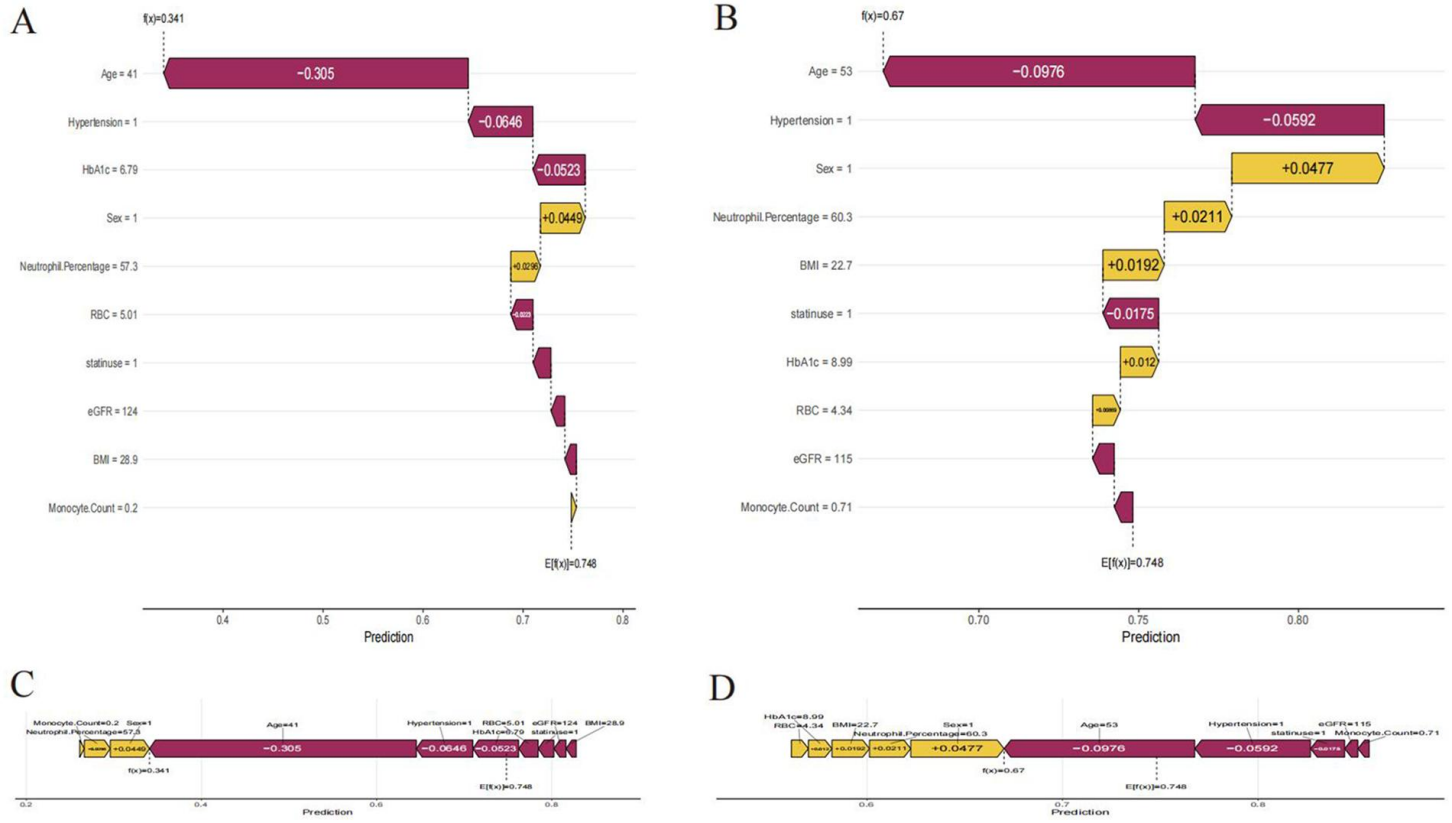
Local model explanation by the SHAP method. Waterfall plot and evolution of risks contributed by each feature for individual patients at low **(A)** or high **(B)** risk of developing carotid plaque: **(A)** represents the individual patients toward the “non-carotid plaque” class, and **(B)** represent the individual patients toward the “carotid plaque” class. SHAP force plots of **(C)** patient 1 (true negative) and **(D)** patient 2 (true-positive). Each patient is represented on the *x*-axis, while the features’ contributions are represented on the *y*-axis; an increased yellow part for each individual patient represents a greater probability toward the decision of “carotid plaque”.

A local explanation demonstrates how specific patient-input data can be used to generate individual predictions. Figures 8A,B presents a case study of a patient with a risk of carotid plaque based on clinical results. The waterfall chart of the Logistic Regression model classified the patient as “no carotid plaque” with a probability of 34.1% (Figure 10A), and classified him as “carotid plaque” with a probability of 67% (Figure 10B). The corresponding force plot (Figures 10C,D) shows the actual measured characteristic values that contribute to these predictions.

### 3.7 Web application development

**Figure 11:**
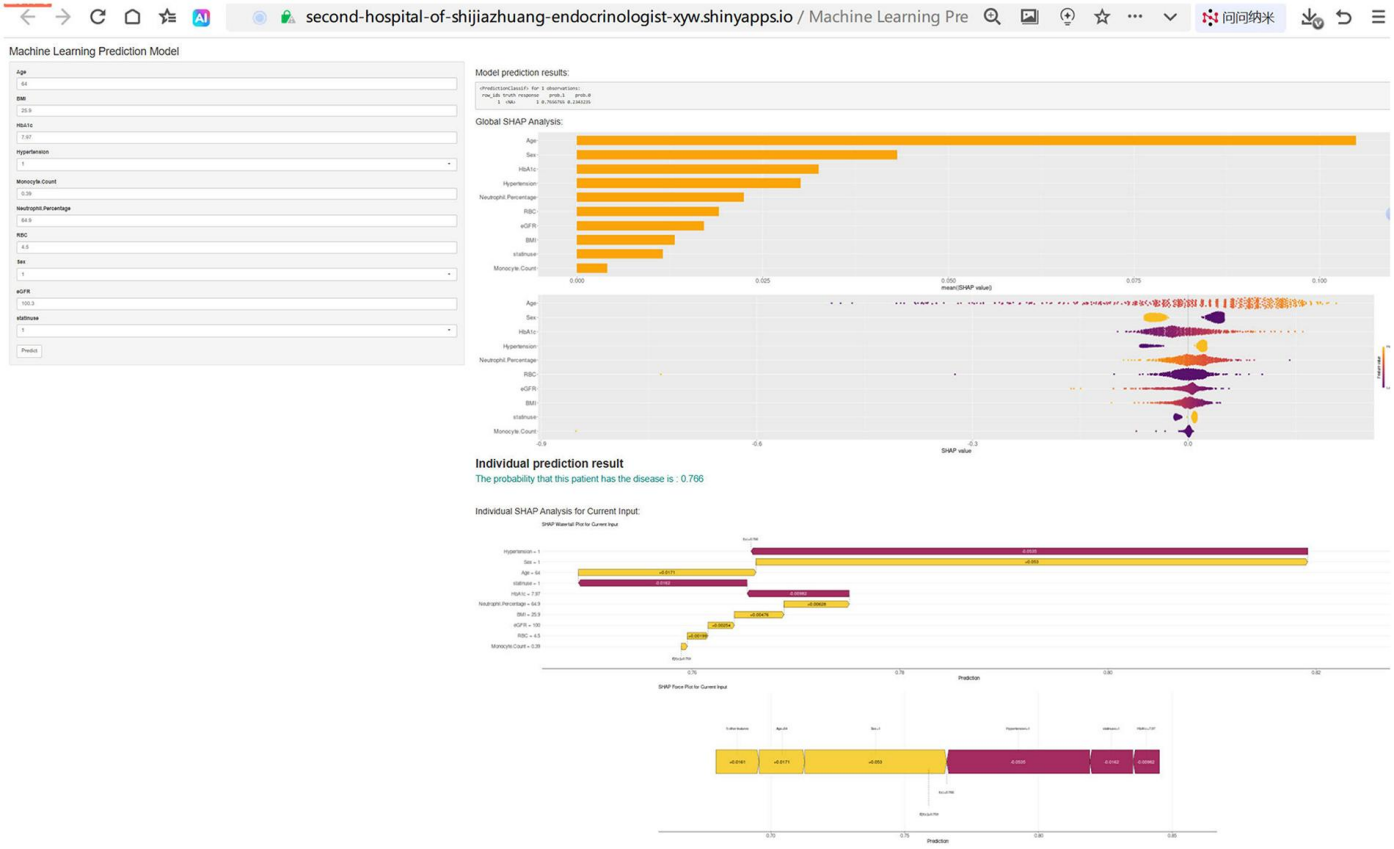
Online computing application presentation of the optimal logistic regression model.

Based on the optimal Logistic Regression model, we developed an easy-to-use online tool that enables primary care physicians to predict the occurrence of carotid plaque in patients with type 2 diabetes undergoing routine visits in real time (https://second-hospital-of-shijiazhuang-endocrinologist-xyw.shinyapps.io/ML_Prediction_Model/, Figure 11). Users input 10 required feature values, and the application automatically calculates the probability of the carotid plaque. Additionally, personalized waterfall charts and force-directed graphs were generated to provide an intuitive explanation of the contribution of each feature to each prediction.

## 4 Disscussion

Our analysis indicated that, in patients with type 2 diabetes, carotid plaque was predominantly associated with older age, higher body mass index, poorer glycemic control (HbA1c), a history of hypertension, increased monocyte counts, a higher neutrophil proportion, red blood cell count, sex, lower eGFR, and statin use. These findings are broadly consistent with those of previous reports (12–16). In the temporal validation data, the model exhibited robust discriminative ability, satisfactory calibration, and favorable clinical utility, supporting its potential as a screening tool for the presence of existing carotid plaque in patients with type 2 diabetes. However, the low specificity observed (approximately 0.24–0.26) warrants careful consideration in clinical implementation, as it may result in a substantial number of false-positive findings in screening settings. A predefined model update strategy, including version control and periodic performance reassessment, has been established to support the long-term clinical utility and reproducibility of the online application.

Historically, the timely detection of carotid lesions has depended on the ultrasound examinations conducted by experienced sonographers. However, in China, the high prevalence of diabetes coupled with under-resourced primary care makes it difficult to deploy specialist sonographers in community clinics, thereby limiting the early surveillance of carotid atherosclerotic plaques among people with diabetes. Machine learning can synthesize routinely collected demographic, laboratory, medical history, and lifestyle data to deliver noninvasive, individualized risk assessments that identify high-risk individuals earlier for lifestyle modification or pharmacotherapy. These models can also concentrate on screening truly high-risk groups, spare unnecessary testing of low-risk individuals, improve screening efficiency, and reduce costs. Cost estimates indicate model-based assessment costs ≈CNY 39 per person vs. ≈CNY 144 for routine carotid ultrasound, a saving of ∼CNY 105 per person extrapolated to 800,000 residents, which corresponds to a theoretical reduction in expenditure of approximately CNY 84 million. Thus, the proposed risk calculator could enable more precise screening and judicious allocation of health care resources.

Using routinely collected clinical variables, we built carotid-plaque risk models using seven machine learning algorithms. Head-to-head evaluations showed that the LR achieved the best overall performance. An LR model with ten key predictors demonstrated stability and clinical applicability. Compared with conventional statistical methods, machine learning can yield higher accuracy when sample sizes are moderate but feature dimensionality is large. However, highly parameterized architectures (e.g., deep neural networks) present a “black-box” interpretability problem because their representational capacity and training dynamics lack a unifying theoretical description. To increase transparency, we applied SHAP to the LR model to quantify each variable's contribution, offering both global importance rankings and individual-level attributions, thereby enhancing interpretability and clinical acceptability (17).

In the present analysis, logistic regression outperformed ensemble methods in both discrimination and calibration. This finding carries methodological significance, as it directly challenges the prevalent assumption in the contemporary medical prediction literature that complex machine learning algorithms inherently surpass traditional statistical models. For this specific clinical prediction task, the simplicity, transparency, and relative robustness of logistic regression to overfitting in the context of moderate sample size likely contributed to its superior performance. The inferior performance of ensemble methods in our analysis may reflect overfitting to noise, instability arising from the relatively limited number of outcome events, or the fact that the underlying predictor-outcome relationships were adequately captured by linear functions. This “negative result” for advanced machine learning techniques is not a limitation of the present study but rather a key contribution: it provides an empirical caution against model selection driven by methodological novelty rather than by demonstrated performance in the relevant clinical context. Deployment of unnecessarily complex and opaque algorithms in settings where simpler models perform equally well or better may hinder clinical adoption and introduce avoidable instability. These findings underscore the importance of conducting rigorous head-to-head comparisons rather than presuming the superiority of any particular model class.

Consistent with prior studies, the variables most strongly associated with prevalent carotid plaque in our models were older age, male sex, higher HbA1c, and a history of hypertension; these associations reflect cross-sectional relationships and do not imply causality or that modification of these variables will necessarily alter plaque incidence or progression. Early recognition of these factors is pivotal for evaluating and preventing plaque formation and consequent cardiovascular events in diabetes. Age carried the greatest weight in feature importance, consistent with the evidence that arterial compliance diminishes with age, facilitating atherogenesis. Large-population studies and a synthesis of evidence have estimated that by 2020, approximately 200 million Chinese individuals have carotid plaques (18). Community ultrasound surveys show that plaque prevalence and burden rise steadily with age, are higher in men, and may appear before the age of 40 in a subset (19, 20). Sex and glycemic exposure ranked second and third, respectively. HbA1c, an index of chronic hyperglycemia, correlates with higher odds of plaque presence and is an imaging marker of vulnerability (21, 22). Mechanistically, persistent hyperglycemia fosters advanced glycation end-product (AGE) accumulation. AGE–RAGE interactions activate NF-*κ*B signaling, induce proinflammatory mediators, upregulate adhesion molecules (VCAM-1, ICAM-1) and chemokines, and promote monocyte adherence and intimal migration, which are key steps in atheroma initiation (23). Elevated HbA1c levels are also associated with reduced or dysfunctional endothelial progenitor cells, impaired vascular repair, and higher inflammatory biomarkers (hs-CRP, IL-6), which stimulate matrix metalloproteinases that degrade the fibrous cap and increase plaque instability (24) (14, 21).

Sex influences clinical presentation and risk of atherosclerosis. In a large T2DM cross-sectional study, plaque–stroke association was stronger in men, whereas carotid IMT correlated more with stroke in women, older patients, and those with longer disease duration (25). Coexisting hypertension markedly increases the risk of advanced glycation end-product (AGE)-receptor for Advanced Glycation End-products(RAGE)-mediated endothelial inflammation, increased shear/wall stress, impaired intimal lipid efflux, and fragile neovessels that favor intraplaque hemorrhage (26–28).

In addition, BMI and white- blood cell count were included in the LR model. Elevated BMI promotes carotid plaque formation by increasing peripheral vascular resistance (29), and early arterial stiffening in obese youth highlights BMI control (30). Epidemiological studies have linked inflammatory markers, including leukocyte count, to carotid plaque development (31, 32), and inflammation may increase plaque risk even in metabolically healthy, normal-weight people (33). Our results are consistent with those of previous studies.

Notably, current statin use emerged as a positive predictor in our model. This finding should not be interpreted as evidence that statins increase carotid plaque risk. Rather, it most likely reflects confounding by indication and reverse causation: patients prescribed statins typically have established dyslipidemia, prior cardiovascular disease, or a higher burden of cardiometabolic risk factors and therefore are more likely to have prevalent atherosclerotic plaques. In our cross-sectional dataset, the statin indicator therefore functions as a marker of elevated baseline cardiovascular risk.

The machine learning algorithm developed in this study showed potential utility in screening for prevalent carotid plaque in patients with type 2 diabetes using routinely collected clinical variables. This approach may assist primary care physicians in identifying individuals at higher risk, potentially enabling more targeted screening strategies. To support clinical interpretation, we propose a risk stratification framework based on predicted probabilities: <25% (low likelihood, routine management), 25%–50% (low-to-moderate, shared decision-making), 50%–75% (moderate-to-high, consider ultrasound and risk factor modification), and >75% (high likelihood, carotid imaging and therapy optimization). This framework may assist clinicians in tailoring screening strategies to individual risk profiles.

We systematically evaluated alternative thresholds beyond the conventional 0.5 cutoff and identified 0.7 as a more balanced operating point for screening, where sensitivity remained acceptable at 0.878 (95% CI: 0.809–0.943) while specificity improved substantially from 0.267 to 0.556 (95% CI: 0.416–0.690), illustrating the inherent trade-off between case detection and false-positive reduction. This deliberate calibration nearly doubled the positive predictive value to 0.798 (95% CI: 0.719–0.872), meaning four of five flagged cases were true positives—a ratio that is arguably acceptable for a non-invasive screening test when the alternative is universal imaging. Future refinement strategies to further improve specificity without compromising sensitivity include incorporating biomarkers (e.g., lipoprotein(a), hs-CRP), adopting a two-stage screening paradigm with confirmatory testing, and restricting application to higher-prevalence subpopulations to leverage Bayes' theorem and enrich pre-test probability.

### 4.1 Strength and limitation

Despite its clinical relevance, this study has several limitations. It used cross-sectional data from a single center, precluding longitudinal inference or absolute incidence estimates and limiting causal and time trend predictions. It must be emphasized that, due to the inherent limitations of the cross-sectional design, this model is not intended to predict the future occurrence or progression of the lesions, but rather to describe the characteristic profile of individuals currently at high risk. Recruitment at a general hospital in northern China restricts external validity; dietary, climatic, and demographic (e.g., southern regions or ethnic minorities) differences may alter the model performance. The sensitivity was modest, risking missed cases if used for screening. A lean predictor set aids feasibility, but may reduce discrimination and generalizability. Improving robustness and clinical utility requires multicenter, diverse cohorts, prospective follow-up, and methodological refinement. Residual confounding and missing data on statin dose, duration, and adherence remain limitations. These factors may bias treatment–outcome associations. Validation was temporal, using a later data from the same institution, so findings show temporal stability but not generalizability across sites. Therefore, the current model should be viewed as a proof-of-concept for identifying prevalent plaque using routine data, and its clinical deployment would require further validation and refinement in prospective, multicenter studies to improve specificity and positive predictive value.

## 5 Conclusion

We developed an interpretable clinical ML model using routine type 2 diabetes data to predicting the presence of plaque as initial risk stratification tool, bridge community diabetes care, and vascular screening for early ASCVD risk stratification.

## Data Availability

The raw data supporting the conclusions of this article will be made available by the authors, without undue reservation.

## Supplementary material

Supplementary Figure S1 Correlation Matrix of Features before feature selection. This figure displays the correlation matrix among the features in the development and validation cohorts, utilizing Pearson's correlation coefficients to assess the linear relationships between variables. The intensity of the colors represents the strength of the correlations, ranging from deep blue (negative correlation) to deep red (positive correlation), with values spanning from -1 to 1. Positive values denote positive correlations, while negative values indicate negative correlations. The diagonal values are all one, representing the perfect correlation of each feature with itself. BMI, body mass index; eGFR, estimated glomerular filtration rate; HbA1c, glycated hemoglobin; RBC, red blood cell count.

Supplementary Figure S2 Correlation matrix of selected features after feature selection in the prediction model for carotid plaque in type 2 diabetes patients. The heatmap displays pairwise Pearson correlation coefficients between the features retained after the feature-selection process. Color intensity and direction indicate the strength and sign of the correlation, with red representing positive correlations and blue representing negative correlations. The diagonal is left blank for clarity. Only features with significant relevance to plaque prediction were included to reduce multicollinearity and enhance the model interpretability. Correlation coefficients range from −1 to +1, as indicated by the color bar. Feature selection was performed using the [mention method, for example, recursive feature elimination, Lasso, or SHAP-based selection] prior to model training. This matrix supports the independence assumption of the selected predictors in the subsequent machine learning model.

Supplementary Figure S3 SHAP dependence plots for the top six features in the logistic regression model predicting carotid plaque in type 2 diabetes patients. SHAP (SHapley Additive exPlanations) dependence plots illustrate how individual features influence the model's output for plaque prediction. Each dot represents one patient. (A) Age (years) vs. SHAP value for plaque prediction. (B) Sex (categorical) vs. SHAP value. (C) HbA1c (%) vs. SHAP value. (D) Hypertension status (categorical) vs. SHAP value. (E) Neutrophil percentage (%) vs. SHAP value. (F) Red blood cell count (RBC, ×10¹²/L) vs. SHAP value. The x-axis shows the actual feature value for each patient; the y-axis indicates the SHAP value, representing the impact on the model's prediction (positive values favor plaque presence, negative values favor absence).

## Glossary

ADA: American Diabetes Association
AGEs: Advanced Glycation End-products
ASCVD: Atherosclerotic Cardiovascular Disease
AUC: Area Under the Curve
AUPRC: Area Under the Precision-Recall Curve
AUROC: Area Under the Receiver Operating Characteristic curve
BMI: Body Mass Index
BUN: Blood Urea Nitrogen
CCA: Common Carotid Artery
CHOL: Total Cholesterol
CREA: Serum Creatinine
CV: Cross-Validation
DCA: Decision Curve Analysis
DBP: Diastolic Blood Pressure
DM: Diabetes Mellitus
ECA: External Carotid Artery
eGFR: Estimated Glomerular Filtration Rate
EPCs: Endothelial Progenitor Cells
HbA1c: Glycated Hemoglobin
Hs-CRP: High-sensitivity C-Reactive Protein
HDL: High-Density Lipoprotein Cholesterol
HGB: Hemoglobin
ICA: Internal Carotid Artery
ICAM-1: Intercellular Adhesion Molecule-1
IL-6: Interleukin-6
IMT: Intima-Media Thickness
LASSO: Least Absolute Shrinkage and Selection Operator
LDL: Low-Density Lipoprotein Cholesterol
LR: Logistic Regression
ML: Machine Learning
MMPs: Matrix Metalloproteinases
NF-*κ*B: Nuclear Factor Kappa-Light-Chain-Enhancer of Activated B Cells
PDW: Platelet Distribution Width
PLT: Platelet Count
PR: Precision-Recall
RAGE: Receptor for Advanced Glycation End-products
RBC: Red Blood Cell Count
RDW-SD: Red Cell Distribution Width-Standard Deviation
ROC: Receiver Operating Characteristic
SBP: Systolic Blood Pressure
SHAP: Shapley Additive Explanations
T2DM: Type 2 Diabetes Mellitus
TG: Triglycerides
UA: Uric Acid
VCAM-1: Vascular Cell Adhesion Molecule-1
VIF: Variance Inflation Factor
WBC: White Blood Cell Count.
