# Supplementary figures and images for: Development and validation of a machine learning-based predictive model for carotid plaque in type 2 diabetes
Source: Front Cardiovasc Med. 2026 Jun 12;13:1801899. doi: 10.3389/fcvm.2026.1801899 (PMC13303130; doi:10.3389/fcvm.2026.1801899)

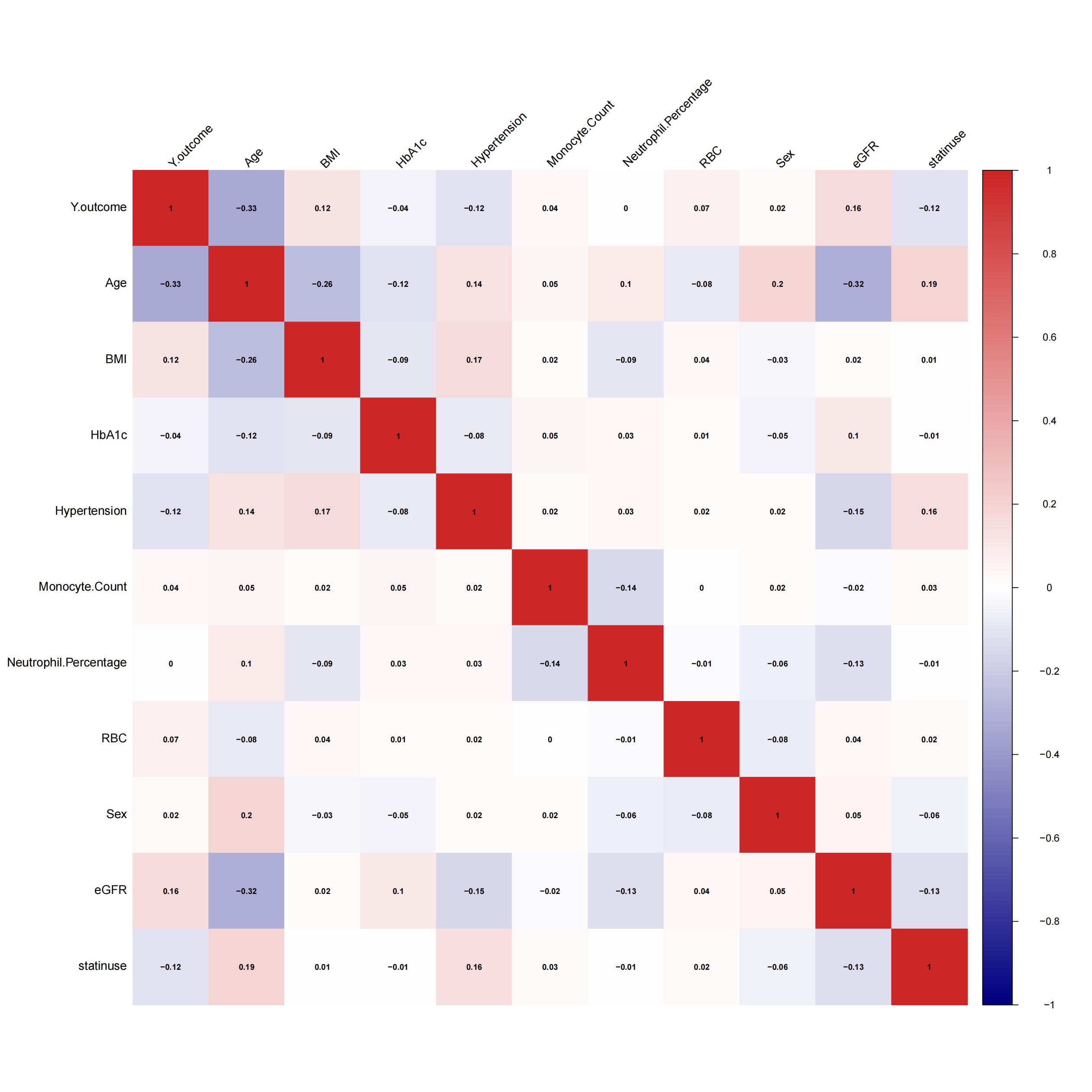

Supplement: Supplementary Figure S1 — Correlation Matrix of Features before feature selection. This figure displays the correlation matrix among the features in the development and validation cohorts, utilizing Pearson's correlation coefficients to assess the linear relationships between variables. The intensity of the colors represents the strength of the correlations, ranging from deep blue (negative correlation) to deep red (positive correlation), with values spanning from -1 to 1. Positive values denote positive correlations, while negative values indicate negative correlations. The diagonal values are all one, representing the perfect correlation of each feature with itself. BMI, body mass index; eGFR, estimated glomerular filtration rate; HbA1c, glycated hemoglobin; RBC, red blood cell count. [file Image1.jpeg]

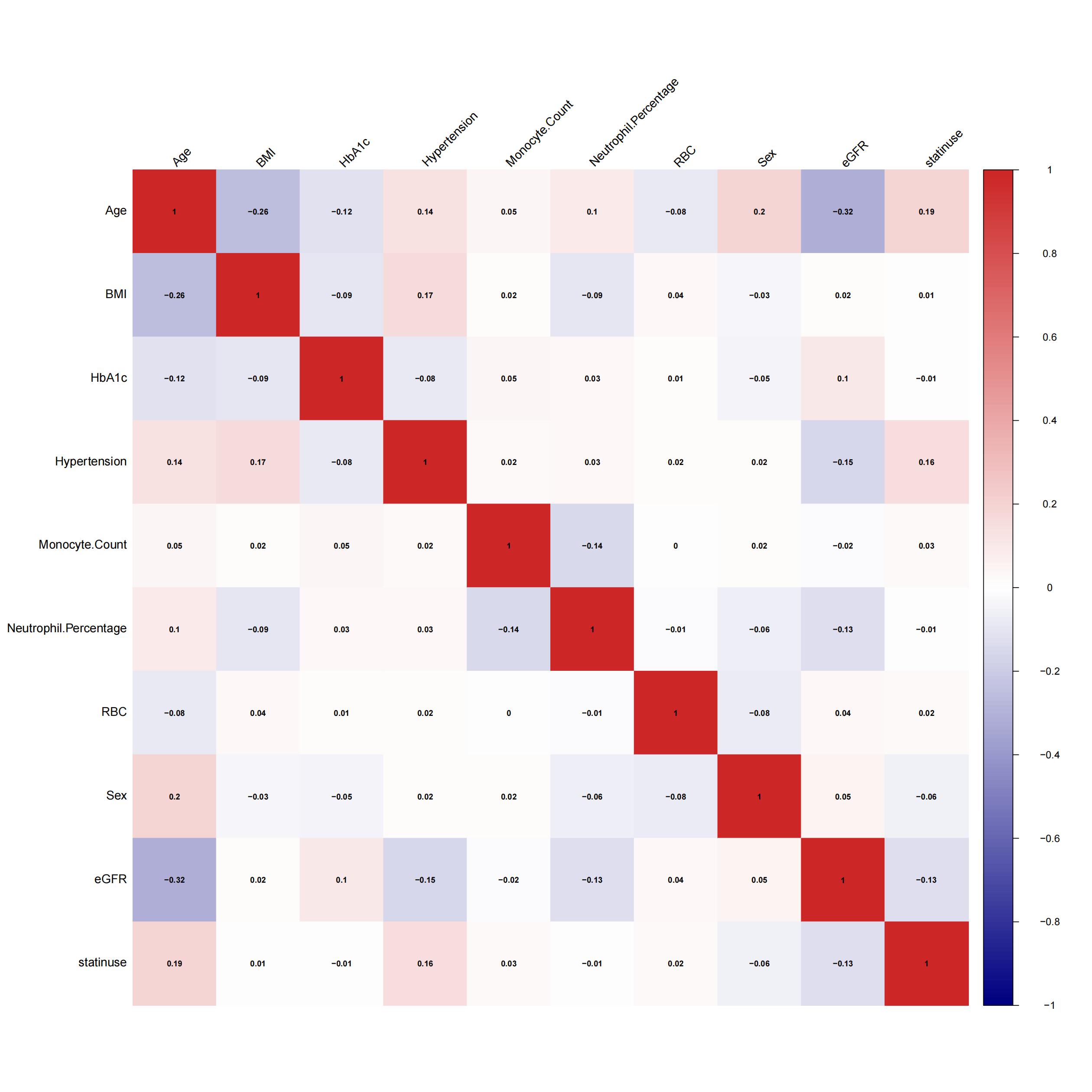

Supplement: Supplementary Figure S2 — Correlation matrix of selected features after feature selection in the prediction model for carotid plaque in type 2 diabetes patients. The heatmap displays pairwise Pearson correlation coefficients between the features retained after the feature-selection process. Color intensity and direction indicate the strength and sign of the correlation, with red representing positive correlations and blue representing negative correlations. The diagonal is left blank for clarity. Only features with significant relevance to plaque prediction were included to reduce multicollinearity and enhance the model interpretability. Correlation coefficients range from −1 to +1, as indicated by the color bar. Feature selection was performed using the [mention method, for example, recursive feature elimination, Lasso, or SHAP-based selection] prior to model training. This matrix supports the independence assumption of the selected predictors in the subsequent machine learning model. [file Image2.jpeg]

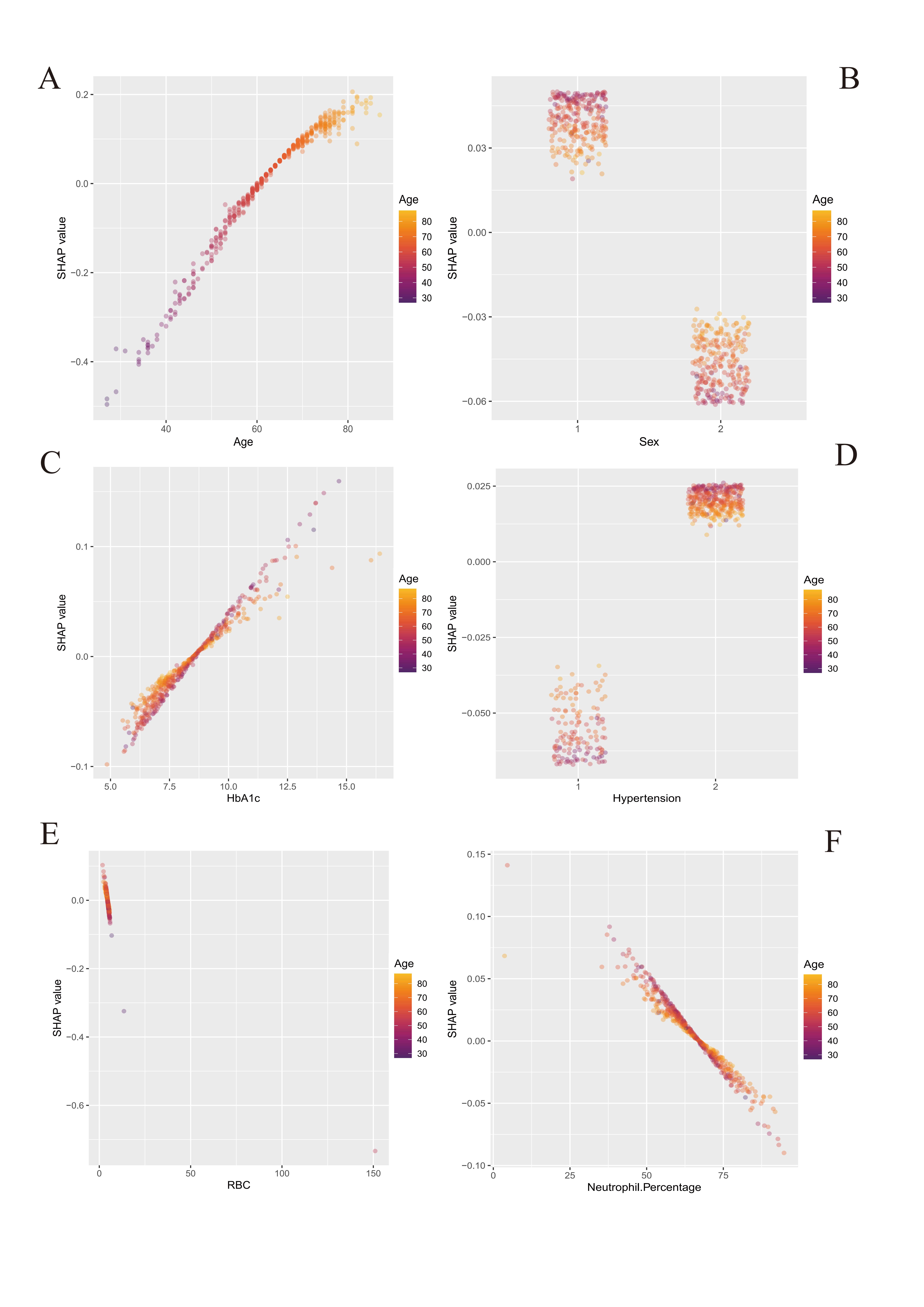

Supplement: Supplementary Figure S3 — SHAP dependence plots for the top six features in the logistic regression model predicting carotid plaque in type 2 diabetes patients. SHAP (SHapley Additive exPlanations) dependence plots illustrate how individual features influence the model's output for plaque prediction. Each dot represents one patient. (A) Age (years) vs. SHAP value for plaque prediction. (B) Sex (categorical) vs. SHAP value. (C) HbA1c (%) vs. SHAP value. (D) Hypertension status (categorical) vs. SHAP value. (E) Neutrophil percentage (%) vs. SHAP value. (F) Red blood cell count (RBC, ×10¹²/L) vs. SHAP value. The x-axis shows the actual feature value for each patient; the y-axis indicates the SHAP value, representing the impact on the model's prediction (positive values favor plaque presence, negative values favor absence). [file Image3.jpeg]
