# Supplementary material for: Development and validation of a machine learning-based predictive model for carotid plaque in type 2 diabetes
Source: Front Cardiovasc Med. 2026 Jun 12;13:1801899. doi: 10.3389/fcvm.2026.1801899 (PMC13303130; doi:10.3389/fcvm.2026.1801899)
Supplement: Supplementary file 4 [file Table1.docx]

**Supplementary Table 1. Definitions and Types of Variables**

| **Category** | **Variable** | **Definition and Variable Type** |
| --- | --- | --- |
| **Demographic Characteristics** | |  |
|  | Sex | Biological sex of the individual (male or female). A binary variable. |
|  | Age | The age of the individual in years. A continuous variable. |
| **Lifestyle Habits** | |  |
|  | Smoking History | Current tobacco smoking status (yes/no). A binary variable. |
|  | Drinking History | Current alcohol consumption status (yes/no). A binary variable. |
| **Clinical Assessments and Vital Signs** | |  |
|  | SBP | Systolic blood pressure (mmHg). A continuous variable. |
|  | DBP | Diastolic blood pressure (mmHg). A continuous variable. |
|  | BMI | Body mass index (kg/m²). A continuous variable. |
| **Medical History** | |  |
|  | Hypertension | Physician-diagnosed hypertension (yes/no). A binary variable. |
|  | DM Duration | Duration of diabetes mellitus (years). A continuous variable. |
|  | Family History of Diabetes | History of diabetes in first-degree relatives (yes/no). A binary variable. |
|  | Family History of Hypertension | History of hypertension in first-degree relatives (yes/no). A binary variable. |
|  | statinuse | Current use of statin medication (yes/no). A binary variable. |
| **Laboratory Examination** | |  |
|  | HbA1c | Glycated hemoglobin (%). A continuous variable. |
|  | WBC | White blood cell count (×10⁹/L). A continuous variable. |
|  | Lymphocyte.Count | Lymphocyte count (×10⁹/L). A continuous variable. |
|  | Neutrophil.Count | Neutrophil count (×10⁹/L). A continuous variable. |
|  | Neutrophil.Percentage | Neutrophil percentage (%). A continuous variable. |
|  | Monocyte.Count | Monocyte count (×10⁹/L). A continuous variable. |
|  | RBC | Red blood cell count (×10¹²/L). A continuous variable. |
|  | HGB | Hemoglobin concentration (g/L). A continuous variable. |
|  | Red.Cell.Distribution.Width.SD | Red cell distribution width - standard deviation (fL). A continuous variable. |
|  | PLT | Platelet count (×10⁹/L). A continuous variable. |
|  | Platelet.Distribution.Width | Platelet distribution width (%). A continuous variable. |
|  | BUN | Blood urea nitrogen (mmol/L). A continuous variable. |
|  | CREA | Serum creatinine (μmol/L). A continuous variable. |
|  | UA | Uric acid (μmol/L). A continuous variable. |
|  | eGFR | Estimated glomerular filtration rate (mL/min/1.73 m²). A continuous variable. |
|  | TG | Triglycerides (mmol/L). A continuous variable. |
|  | CHOL | Total cholesterol (mmol/L). A continuous variable. |
|  | HDL | High-density lipoprotein cholesterol (mmol/L). A continuous variable. |
|  | LDL | Low-density lipoprotein cholesterol (mmol/L). A continuous variable. |
|  | H.CRP | High-sensitivity C-reactive protein (mg/L). A continuous variable. |
|  |  |  |
|  | carotid plaque | Cases were defined as whether participants were diagnosed with carotid plaque by carotid ultrasound. The diameters and IMT of the distal common carotid artery, the carotid bulb, and the proximal internal carotid artery were measured within 1–1.5 cm below the level of the bifurcation of the participant’s internal and external carotid arteries by an experienced physician to observe the presence of atherosclerotic plaque.(参考文献：doi: 10.3389/fcvm.2025.1490961) |

Abbreviations: BMI, body mass index; eGFR, estimated glomerular filtration rate; HbA1c, glycated hemoglobin; RBC, red blood cell count;
