# Supplementary material for: Development and validation of a machine learning-based predictive model for carotid plaque in type 2 diabetes
Source: Front Cardiovasc Med. 2026 Jun 12;13:1801899. doi: 10.3389/fcvm.2026.1801899 (PMC13303130; doi:10.3389/fcvm.2026.1801899)
Supplement: Supplementary file 5 [file Table2.docx]

**Supplementary Table 2. Baseline and Procedural Characteristics of Patients**

| \| **Variable** \| **Model Development and Validation**  **(n = 1601)** \| **Test Data (n = 687)** \| **Overall**  **(n = 2288)** \| ***p*-value** \| \| --- \| --- \| --- \| --- \| --- \| \| Carotid Plaque, n (%) \|  \|  \|  \| 0.979 \| \| 0 (Negative) \| 400 (25.0) \| 172 (25.0) \| 572 (25.0) \|  \| \| 1 (Positive) \| 1201 (75.0) \| 515 (75.0) \| 1716 (75.0) \|  \| \| Demographics and Clinical Variables \|  \|  \|  \|  \| \| Age, years, mean ± SD \| 63.2 ± 11.5 \| 62.4 ± 11.9 \| 63.0 ± 11.6 \| 0.108 \| \| BMI, kg/m², mean ± SD \| 26.2 ± 3.9 \| 26.8 ± 13.1 \| 26.4 ± 7.9 \| 0.126 \| \| Sex, n (%) \|  \|  \|  \| 0.523 \| \| Male \| 853 (53.3) \| 376 (54.7) \| 1229 (53.7) \|  \| \| Female \| 748 (46.7) \| 311 (45.3) \| 1059 (46.3) \|  \| \| Laboratory and Comorbidities \|  \|  \|  \|  \| \| HbA1c, %, mean ± SD \| 8.4 ± 1.9 \| 8.4 ± 1.9 \| 8.4 ± 1.9 \| 0.875 \| \| Hypertension, n (%) \|  \|  \|  \| 0.654 \| \| No \| 479 (29.9) \| 212 (30.9) \| 691 (30.2) \|  \| \| Yes \| 1122 (70.1) \| 475 (69.1) \| 1597 (69.8) \|  \| \| Monocyte Count, ×10⁹/L, median (IQR) \| 0.4 (0.3, 0.5) \| 0.4 (0.3, 0.5) \| 0.4 (0.3, 0.5) \| 0.428 \| \| Neutrophil Percentage, %, mean ± SD \| 65.1 ± 10.7 \| 66.1 ± 10.8 \| 65.4 ± 10.7 \| 0.031 \| \| Red Blood Cells (RBC), ×10¹²/L, median (IQR) \| 4.5 (4.1, 4.9) \| 4.5 (4.1, 4.9) \| 4.5 (4.1, 4.9) \| 0.757 \| \| eGFR, mL/min/1.73 m², mean ± SD \| 107.3 ± 42.3 \| 106.9 ± 39.7 \| 107.2 ± 41.5 \| 0.826 \| \| Statin Use, n (%) \|  \|  \|  \| 0.539 \| \| No \| 670 (41.8) \| 297 (43.2) \| 967 (42.3) \|  \| \| Yes \| 931 (58.2) \| 390 (56.8) \| 1321 (57.7) \|  \| | | |  | | |
| --- | --- | --- | --- | --- | --- | --- | --- | --- | --- | --- | --- | --- | --- | --- | --- | --- | --- | --- | --- | --- | --- | --- | --- | --- | --- | --- | --- | --- | --- | --- | --- | --- | --- | --- | --- | --- | --- | --- | --- | --- | --- | --- | --- | --- | --- | --- | --- | --- | --- | --- | --- | --- | --- | --- | --- | --- | --- | --- | --- | --- | --- | --- | --- | --- | --- | --- | --- | --- | --- | --- | --- | --- | --- | --- | --- | --- | --- | --- | --- | --- | --- | --- | --- | --- | --- | --- | --- | --- | --- | --- | --- | --- | --- | --- | --- | --- | --- | --- | --- | --- | --- | --- | --- | --- | --- | --- | --- | --- | --- | --- | --- | --- | --- | --- | --- |
| Abbreviations: BMI, body mass index; eGFR, estimated glomerular filtration rate; HbA1c, glycated hemoglobin; IQR, interquartile range; RBC, red blood cell count; SD, standard deviation.  Categorical variables are presented as frequencies (%). For continuous variables, non-normally distributed data are presented as median (interquartile range), and normally distributed data are presented as mean (standard deviation). |  |  | |  |  |
