# Supplementary material for: Development and validation of a machine learning-based predictive model for carotid plaque in type 2 diabetes
Source: Front Cardiovasc Med. 2026 Jun 12;13:1801899. doi: 10.3389/fcvm.2026.1801899 (PMC13303130; doi:10.3389/fcvm.2026.1801899)
Supplement: Supplementary file 6 [file Table3.docx]

**Supplementary Table 3. Performance Metrics of Machine Learning Model Benchmarking on Training and Validation Sets**

| **Metric** | **AdaBoostM1** | **gbm** | **lightgbm** | **logistic** | **naive_bayes** | **ranger** | **xgboost** |
| --- | --- | --- | --- | --- | --- | --- | --- |
| **auc** | 0.675 | 0.709 | 0.647 | 0.73 | 0.672 | 0.684 | 0.661 |
| **acc** | 0.75 | 0.761 | 0.738 | 0.773 | 0.753 | 0.761 | 0.728 |
| **bbrier** | 0.175 | 0.167 | 0.194 | 0.161 | 0.229 | 0.171 | 0.211 |
| **fbeta** | 0.85 | 0.857 | 0.836 | 0.863 | 0.856 | 0.856 | 0.828 |
| **prauc** | 0.841 | 0.861 | 0.831 | 0.875 | 0.843 | 0.843 | 0.836 |
| **precision** | 0.77 | 0.778 | 0.786 | 0.79 | 0.761 | 0.78 | 0.786 |
| **recall** | 0.951 | 0.953 | 0.895 | 0.95 | 0.98 | 0.95 | 0.878 |
| **sensitivity** | 0.951 | 0.953 | 0.895 | 0.95 | 0.98 | 0.95 | 0.878 |
| **specificity** | 0.143 | 0.186 | 0.268 | 0.246 | 0.077 | 0.195 | 0.283 |
| **bacc** | 0.547 | 0.57 | 0.582 | 0.598 | 0.529 | 0.572 | 0.58 |
| **ce** | 0.25 | 0.239 | 0.262 | 0.227 | 0.247 | 0.239 | 0.272 |
| **dor** | 11.6 | 6.118 | 3.215 | 6.304 | 5.552 | 4.913 | 2.951 |
| **fn** | 11.6 | 11.4 | 25.2 | 12 | 4.8 | 12.2 | 29.4 |
| **fnr** | 0.049 | 0.047 | 0.105 | 0.05 | 0.02 | 0.05 | 0.122 |
| **fomr** | 0.358 | 0.426 | 0.54 | 0.38 | 0.434 | 0.436 | 0.579 |
| **fp** | 68.4 | 65.2 | 58.8 | 60.6 | 74.2 | 64.4 | 57.8 |
| **fpr** | 0.857 | 0.814 | 0.732 | 0.754 | 0.923 | 0.805 | 0.717 |
| **logloss** | 0.532 | 0.511 | 0.64 | 0.501 | 1.182 | 0.526 | 0.613 |
| **npv** | 0.642 | 0.574 | 0.46 | 0.62 | 0.566 | 0.564 | 0.421 |
| **ppv** | 0.77 | 0.778 | 0.786 | 0.79 | 0.761 | 0.78 | 0.786 |
| **tn** | 11.6 | 14.8 | 21.2 | 19.4 | 5.8 | 15.6 | 22.2 |
| **tnr** | 0.143 | 0.186 | 0.268 | 0.246 | 0.077 | 0.195 | 0.283 |
| **tp** | 228.6 | 228.8 | 215 | 228.2 | 235.4 | 228 | 210.8 |
| **tpr** | 0.951 | 0.953 | 0.895 | 0.95 | 0.98 | 0.95 | 0.878 |

The models evaluated include AdaBoostM1(Adaptive Boosting M1), Gradient Boosting Machine (gbm), Light Gradient Boosting Machine (lightgbm), Logistic Regression (logistic), Naïve Bayes(naive_bayes), Random Forest (ranger), and eXtreme Gradient Boosting (xgboost).

Performance metrics: AUC (Area Under the Curve), ACC (Accuracy), Brier (Brier Score), F-Beta (F-Beta Score), PRAUC (Precision-Recall Area Under the Curve), Precision, Recall, Sensitivity, Specificity, BACC (Balanced Accuracy), CE (Cross Entropy), DOR (Diagnostic Odds Ratio), FN (False Negatives), FNR (False Negative Rate), FOMR (False Omission Rate), FP (False Positives), FPR (False Positive Rate), LogLoss (Logarithmic Loss), NPV (Negative Predictive Value), PPV (Positive Predictive Value), TN (True Negatives), TNR (True Negative Rate), TP (True Positives), TPR (True Positive Rate).
