# Supplementary material for: Development and validation of a machine learning-based predictive model for carotid plaque in type 2 diabetes
Source: Front Cardiovasc Med. 2026 Jun 12;13:1801899. doi: 10.3389/fcvm.2026.1801899 (PMC13303130; doi:10.3389/fcvm.2026.1801899)
Supplement: Supplementary file 7 [file Table4.docx]

Supplementary Table 4. Performance metrics of the model at classification thresholds of 0.5 and 0.7 in the temporal validation data.

| Metric | Threshold 0.5 (95% CI) | Threshold 0.7 (95% CI) |
| --- | --- | --- |
| Sensitivity | 0.978 (0.944–1.000) | 0.878 (0.809–0.943) |
| Specificity | 0.267 (0.155–0.413) | 0.556 (0.416–0.690) |
| PPV | 0.727 (0.640–0.804) | 0.798 (0.719–0.872) |
| NPV | 0.857 (0.667–1.000) | 0.694 (0.560–0.842) |
| Youden index | 0.244 (0.123–0.393) | 0.433 (0.286–0.578) |
| MCC | 0.378 (0.225–0.534) | 0.462 (0.316–0.605) |
| Balanced accuracy | 0.622 (0.562–0.697) | 0.717 (0.643–0.789) |
| F1-score | 0.834 (0.776–0.884) | 0.836 (0.777–0.887) |
| Accuracy | 0.741 (0.667–0.815) | 0.770 (0.696–0.837) |
| ROC-AUC | 0.758 (0.670–0.843) | 0.758 (0.670–0.843) |
| Brier score | 0.183 (0.143–0.229) | 0.183 (0.143–0.229) |

Abbreviations: PPV, positive predictive value; NPV, negative predictive value; MCC, Matthews correlation coefficient; AUC-ROC, area under the receiver operating characteristic curve; CI, confidence interval.

Note: Data are presented as estimate (95% CI). ‡Threshold-independent metrics. The 0.7 threshold was selected based on the Youden index to optimize the sensitivity–specificity trade-off for screening implementation.
